# Homology Model of a Catalytically Competent Bifunctional Rel Protein

**DOI:** 10.3389/fmolb.2021.628596

**Published:** 2021-02-03

**Authors:** Monica Civera, Sara Sattin

**Affiliations:** Department of Chemistry, Università Degli Studi di Milano, Milan, Italy

**Keywords:** bacterial persisters, (p)ppGpp, Rel_Seq_, RelP, homology modeling, chimera, molecular dynamics

## Abstract

Bacteria have developed different bet hedging strategies to survive hostile environments and stressful conditions with persistency being maybe the most elegant yet still poorly understood one. Persisters’ temporary tolerance to antibiotic treatment hints at their role not only in chronic and recurrent infections but also in the insurgence of resistant strains. Therefore, hampering persisters formation might represent an innovative strategy in the quest for new effective antimicrobial compounds. Among the molecular mechanisms postulated for the persister phenotypic switch, we decided to focus our attention on the stringent response and, in particular, on the upstream triggering step that is the accumulation of guanosine tetra- and pentaphosphate, collectivity called (p)ppGpp. Intracellular levels of (p)ppGpp are regulated by a superfamily of enzymes called RSH (RelA/SpoT homologue) that are able to promote its synthesis *via* pyrophosphate transfer from an ATP molecule to the 3’ position of either GDP or GTP. These enzymes are classified based on the structural domain(s) present (only synthetase, only hydrolase, or both). Here we present our work on Rel_*Seq*_ (from *S. equisimilis*), still the only bifunctional Rel protein for which a GDP-bound “synthetase-ON” structure is available. Analysis of the synthetase site, occupied only by GDP, revealed a partially active state, where the supposed ATP binding region is not conformationally apt to accommodate it. In order to achieve a protein model that gets closer to a fully active state, we generated a chimera structure of Rel_*Seq*_ by homology modeling, starting from the crystal structure of the catalytically competent state of RelP, a smaller, single-domain, Rel protein from *S. aureus*. Molecular dynamics simulations allowed verifying the stability of the generated chimera model. Virtual screening and ligand design studies are underway.

## Introduction

Persistence is a very elegant bet hedging strategy adopted by bacteria to survive hostile conditions, such as nutrient starvation and antibiotic treatment (Lewis, 2010; Maisonneuve and Gerdes, 2014). This temporary antibiotic-tolerant phenotype plays a starring role in the difficult treatment and eradication of chronic and recurrent infections (Conlon et al., 2015) and possibly in the insurgence of actual antimicrobial resistance (Cohen et al., 2013; Barrett et al., 2019).

In order to maximize survival chances, the molecular mechanisms underlying persisters formation are likely multiple, complementary, and have not been fully elucidated yet. One of the first hypothesis was the activation of the SOS response with increased levels of toxin/antitoxin (TA) transcripts (Dorr et al., 2010), followed by in depth studies on the TA systems of the gram negative *E. coli* that unfortunately had been marred by phage contamination (Harms et al., 2017) and therefore discarded. Studies on the gram positive *S. aureus* showed instead that a low energy state due to ATP depletion was associated to persisters formation (Conlon et al., 2016), a mechanism that could play an important role in *E. coli* as well (Shan et al., 2017). Intrigued by the role played by the stringent response, a stress-induced signalling cascade in bacterial survival (Irving et al., 2020), we decided to focus our attention on the triggering event of the cascade, i.e. the accumulation within the cell of guanosine tetra- or pentaphosphate, collectively called (p)ppGpp and often referred to simply as alarmone (Kushwaha et al., 2019). (p)ppGpp is an histrionic second messenger that plays pleiotropic effects in bacterial cells and has been directly linked to the insurgence of persisters and antimicrobial resistance (Wu et al., 2010;Kanjee et al., 2012;Liu et al., 2015;Kamarthapu et al., 2016;Dutta et al., 2019;Diez et al., 2020). 

The synthesis and hydrolysis of (p)ppGpp are regulated by a superfamily of enzymes called RSH (RelA/SpoT Homologue), or Rel proteins, that can be divided in two main classes based on their domain composition and size: long and short RSHs (Atkinson et al., 2011; Ronneau and Hallez, 2019). Long RSH proteins share a multidomain architecture carrying an N-terminal hydrolase domain (HD) linked to a synthetase domain (SYNTH), equipped with a C-terminal regulatory region that responds to environmental stimuli. The catalytic domains can be both functional, as in the case of SpoT, or one of them can be catalytically inactive, as in the case of RelA. On the other hand, short RSHs present only either the SYNTH (SAS, small alarmone synthetase) or the HD domain (SAH, small alarmone hydrolase).

Gaining control of the enzymatic activity of these proteins using chemical probes would allow to dissect the role played by the accumulation of (p)ppGpp in the insurgence of antibiotic tolerant bacterial persisters. This is the ultimate goal of our project, which requires structural models of the enzymes for rational design of the probes. We decided to focus our attention on long bifunctional RSH proteins due to the relevance of their role in dangerous pathogens such as *Mycobacteria* (Dahl et al., 2003; Vilcheze and Jacobs, 2019). When we started our work, only two long bifunctional Rel protein structures were available: Rel_*Seq*_ (from *Streptococcus dysgalactiae subsp. equisimilis*)*,* the first long RSH for which a *holo* X-ray crystal structure became available (1VJ7. pdb) (Hogg et al., 2004), and Rel_*Mtb*_, for which an *apo* NTD structure was reported in 2017 (Singal et al., 2017). Only very recently, *i.e.* in the last few months, two additional long RSH structure have been reported, namely Rel_*Tt*_ (6S2U.pdb, 6S2T.pdb, and 6S2V.pdb from *T. thermophilus*) (Tamman et al., 2020) and *Bs*Rel (6YXA.pdb and 6HTQ.pdb from *B. subtilis*). Most of these structures are unfortunately either in the apo form, in a post-catalytic state (from the synthetase point of view), or in a hydrolase-ON conformation. The only potentially synthetase-ON structure (i.e. 6HTQ.pdb) comes from a cryo-EM experiment with RelA bound to the stalled ribosome. Unfortunately, the flexibility observed by the authors for the NTD, where the synthetase domain is located, does not allow gathering any insightful structural information regarding the synthetase site.

In light of this information, the need for a detailed structural model of a catalytically competent synthtetase-ON Rel is even more impelling. As a structural model, we therefore adopted Rel_*Seq*_ (from *Streptococcus dysgalactiae subsp. equisimilis*)*,* the only long RSH for which a putative (substrate bound) synthetase-ON conformation was available (1VJ7. pdb, chain A) (Hogg et al., 2004). In order to facilitate crystallization, the construct was truncated downstream of the SYNTH domain and therefore lacks the C-terminal regulatory domain (residues 386–739).

**FIGURE 1:**
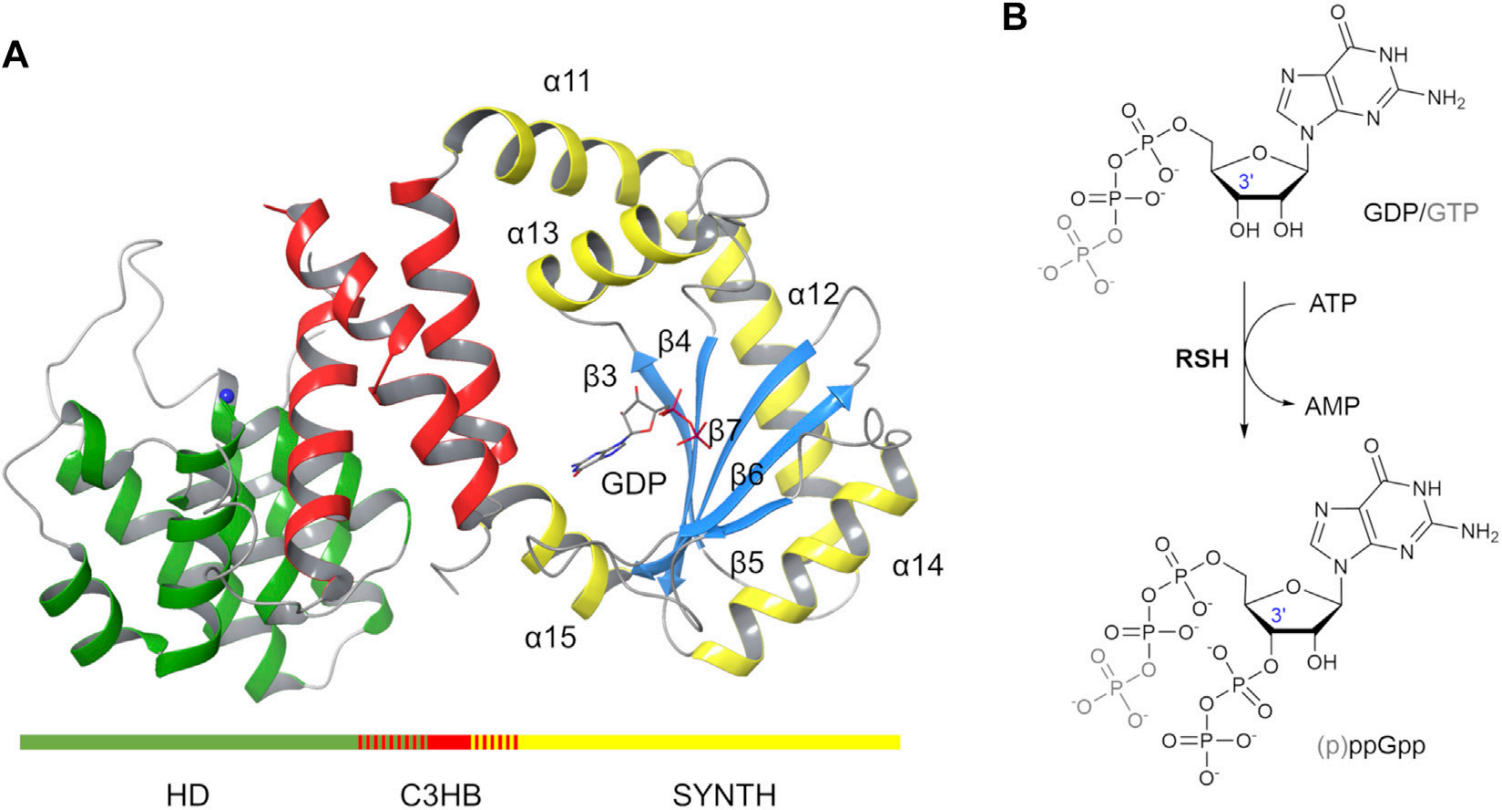
Rel_*Seq*_ X-ray crystal structure and the (p)ppGpp synthesis reaction it catalyses. **(A)** Rel_*Seq*_ crystal structure (1VJ7. pdb, chain A, residues 5–341 are shown) in complex with GDP and Mn^2+^ (blue sphere). **(B)** p)ppGpp is obtained by enzymatic transfer of a PP group from ATP to the 3′-OH group of either GDP or GTP.

From a structural point of view, the N-terminal HD domain of Rel_*Seq*_ is formed by a bundle of α-helices (residues 5–159, Figure 1A, green) while the SYNTH domain (residues 176–371, Figure 1A, yellow and light blue) consists of a 5-stranded β-sheet (β3- β7) surrounded by five helices (α11- α15). A partially overlapping 3-helix bundle links the two catalytic domains (C3HB, Rel_*Seq*_ residues135–195, Figure 1A, red). In the Rel_*Seq*_ crystal structure, it was also possible to locate the substrate molecule GDP in the synthetase site and a Manganese ion in the hydrolase one.

From a catalytic point of view, the synthesis reaction mechanism was initially postulated based on the domain similarity with the DNA polymerase β (Hogg et al., 2004): a Mg^2+^-dependent transfer of the βγ-pyrophosphate of an ATP molecule onto the ribose 3’ hydroxyl group of either GTP or GDP results in pppGpp or ppGpp, respectively (Figure 1B). Interestingly, Rel_*Seq*_ crystal structure presented two distinct conformations that led to postulate the existence of two different catalytic states, one switched toward the synthesis (1VJ7. pdb, chain A, Figure 1A) and the other toward the hydrolysis (1VJ7. pdb, chain B) (Hogg et al., 2004). The conformation labeled synthetase-ON (Figure 1A) holds a GDP substrate molecule bound into the SYNTH active site but lacks both the pyrophosphate donor ATP and the Mg^2+^ co-factor. Additionally, the SYNTH active site of Rel_*Seq*_ is noticeably lacking the appropriate space to allocate ATP in the orientation required by the postulated mechanism, and the putative catalytic residues (D264 and E323) are far away from the substrate GDP. We therefore posited that the crystal structure reported as *synthetase-ON* was really only partially *ON* (i.e. not fully functional) and required further conformational changes to become catalytically competent. As we were writing this paper, a second structure of a bifunctional RSH enzyme isolated from *T. thermophilus* (Rel_*Tt*_) was released in a synthetase post-catalytic active state(open, 6S2U.pdb) and an active hydrolase state (closed, 6S2T.pdb) (Tamman et al., 2020). The open state contains AMP and ppGpp bound in the SYNTH domain, whereas the closed state has ppGpp bound in the HD domain.

The recently deposited structures of Rel_*Tt*_ confirm our hypothesis that the partial occupancy of the SYNTH active site of Rel_*Seq*_ more likely represents an intermediate state, closer to the enzyme resting state than to a fully active catalytic conformation (Tamman et al., 2020).

In our effort to build a more accurate model for the long bifunctional Rel_*Seq*_ we benefitted from the insights provided by the X-ray crystal structures recently published for two SASs, i.e. RelP (*Staphylococcus aureus*) and RelQ (*Bacillus subtilis*) (Manav et al., 2018; Steinchen et al., 2018).

**FIGURE 2:**
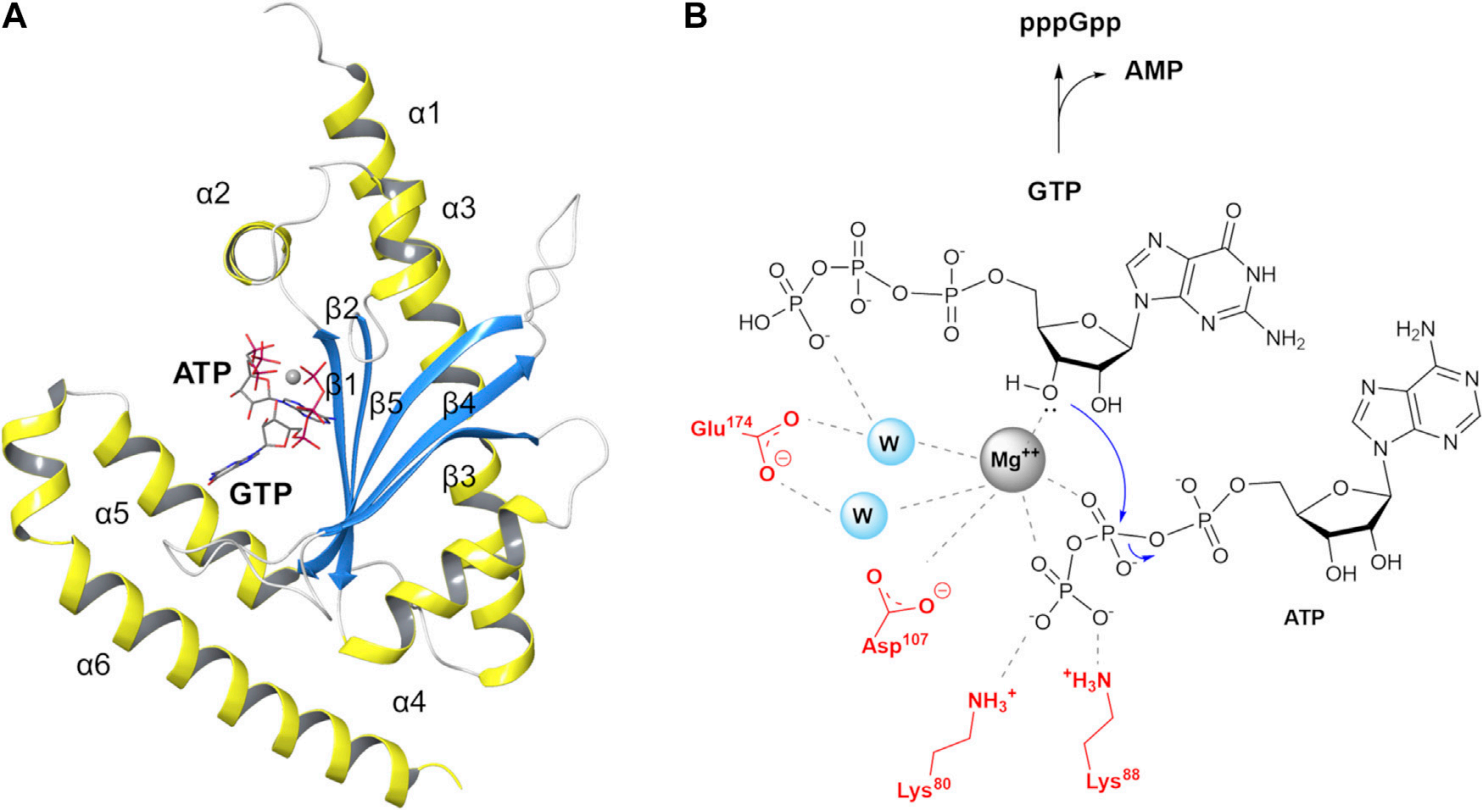
*S. aureus* RelP small alarmone synthetase and its postulated reaction mechanism **(A)** RelP pre-catalytic state (6EWZ.pdb) in complex with GTP, AMP-CPP and Mg^2+^ (gray sphere). **(B)** Proposed reaction mechanism for pppGpp synthesis.

In particular, Manav and co-workers were able to obtain a pre-catalytic complex of RelP bound to its substrate GTP and a non-hydrolysable ATP analogue (AMP-CPP, α,β-methyleneadenosine 5′-triphosphate) coordinated to a Mg^2+^ ion (6EWZ.pdb, Figure 2A) (Manav et al., 2018). This pre-catalytic RelP complex closely resembles the post-catalytic state of the bifunctional Rel_*Tt*_. RelP substrate and Rel_*Tt*_ product nicely overlap, tracing a reproducible interaction network with conserved key residues (Manav et al., 2018; Tamman et al., 2020). These structures constituted an ideal template to build a hybrid (chimera) Rel_*Seq*_-RelP model.

In RelP pre-catalytic state, Mg^2+^ is coordinated to the β- and γ-phosphate groups of AMP-CPP and to the 3′-OH of GTP, thus plausibly priming the latter for the nucleophilic attack on β-phosphate group (Figure 2B). In this pre-transition state geometry, two highly conserved acidic residues, E174 and D107, complete the coordination sphere around Mg^2+^ while the two side chains of K80 and K88 stabilize the negative charge on the terminal phosphate.

**FIGURE 3:**
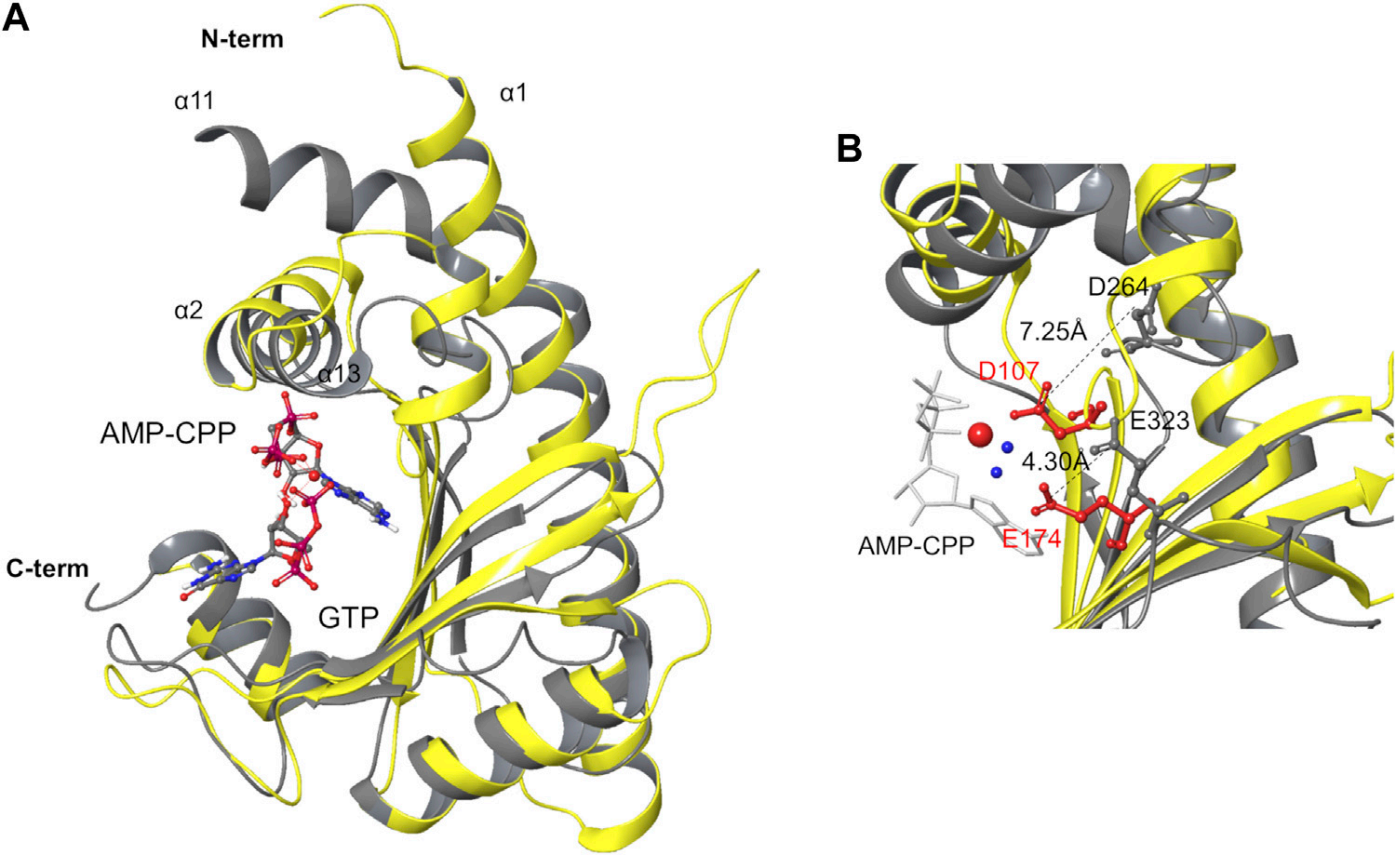
Structure superimposition of the SYNTH domains of Rel_*Seq*_ and RelP. **(A)** Superimposition of Rel_*Seq*_ SYNTH domain (gray ribbons, 1VJ7. pdb chain A, residues 197–341, with GDP ligand) and RelP (yellow ribbons, 6EWZ.pdb, chain A, residues 29–188). **(B)** Catalytic residues of both enzymes are shown (D264, E323 of Rel_*Seq*_ in gray, D107, E174 of RelP in red). Distances between the side chains are reported as calculated on the C atoms of the carboxylate groups. Mg^2+^ is shown as a red sphere with the two coordinated waters in blue, AMP-CPP is in white tube representation while GTP is omitted for clarity.

Comparing the structures of pre-catalytic RelP and of the SYNTH site of Rel_*Seq*_ in the reported *synthetase-ON* state, the argument for a conformational rearrangement of Rel_*Seq*_ required to reach a catalytically competent state becomes even more compelling. Indeed, Rel_*Seq*_ catalytic residues D264 and E323 are not properly oriented relative to the reactive 3’ position of GDP and the orientation of helix α13 prevents ATP from entering into the catalytic site (Figure 3).

Thus, a more realistic model of the Rel_Seq_ *synthetase-ON* state could be built as a homology model of the Rel_*Seq*_ SYNTH domain based on pre-catalytic RelP structure (6EWZ.pdb), which could then be fused to Rel_*Seq*_ HD domain, generating this way a chimera model of the full protein. We here report the generation of such chimera. The construct obtained is stable and, thanks to the rearrangement of the catalytic residues, the ATP molecule is now able to fit into the catalytic site, properly coordinated to the metal and to the key residues of the pocket. This model will be used for ligand design and screening calculations.

## Results and Discussion

### Construction of Rel_*Seq*_–RelP Chimera Models

#### Rel_Seq_–RelP SYNTH Domains Comparison

Rel_*Seq*_ (1VJ7. pdb, chain residues 5–341) and RelP (pre-catalytic state, 6EWZ.pdb, chain A, residues 68–188) crystal structures were prepared using the Protein Preparation Wizard available in Maestro 2018–1 (Maestro, RRID:SCR_016748) (PROPKA at pH = 7 ± 2), and the ligands (GDP, GTP and AMP-CPP) protonation states were determined using Epik (pH 7 ± 2) (Shelley et al., 2007). The most stable fully deprotonated ligand form was chosen for each compound (resulting in a net charge of −4 for AMP-CPP and GTP, and of −3 for GDP).

RelP and Rel_*Seq*_ SYNTH domains were aligned based on their secondary structure elements (RMSD = 2.96 Å, calculated on backbone atoms, Figure 3A) and compared. The catalytic cores share an antiparallel β-sheet (strands β3–β7 in Rel_*Seq*_, Figure 1A, and β1–β5 in RelP, Figure 2A) surrounded by α-helices (α1–α4 in RelP and α12–α14 in Rel_*Seq*_), but they differ for the orientation of the α-helix involved in ATP binding. Indeed, forming an angle of ca. 16° relative to α2 (RelP), the orientation of α13 prevents the accommodation of ATP into Rel_*Seq*_ active site. The orientation of this helix seems to be critical for the activation of the enzyme, as confirmed by the similar orientation of α2 of RelP (pre-catalytic state) and the corresponding α13 of Rel_*Tt*_ (post-catalytic state) (Tamman et al., 2020). Another critical difference is the position of the two key catalytic residues: while D107 and E174 in RelP are ideally placed to coordinate the Mg^2+^ ion cofactor and promote the enzymatic reaction, in the X-ray Rel_Seq_ structure the corresponding D264 and E323 side-chains are tilted away from the Mg^2+^ ion putative position, i.e. the reaction center. The position of the corresponding catalytic residues in the Rel_*Tt*_ post-catalytic structure is similar to RelP pre-catalytic state, thus indicating that a conformation change seems to be necessary for the activation of Rel_*Seq*_. In particular, the glutamic acid residue of all three proteins is located in a rigid secondary structure element (E174 in RelP-β5, E323 in Rel_*Seq*_-β7 and E345 in Rel_*Tt*_-β7, respectively) and its interaction with the Mg^2+^ ion is mediated in RelP by two water molecules. The distance measured between the substrate 3′-O(H) and the Glu-Cδ (5.45 Å in RelP vs 6.53 Å in Rel_*Seq*_) suggests that only a small shift would be required for E323 in Rel_*Seq*_ to become effective. A different situation is observed for the catalytic aspartic acid residues, inserted in a more mobile loop. In RelP, the 3′-O(H) - D107-Cγ distance is already optimal (5.4 Å, Mg-mediated interaction), while in Rel_*Seq*_ the corresponding 3′-O(H)–D264-Cγ distance is much higher (10.4 Å), highlighting that a major conformational change is required. Additional differences between the two SYNTH domains depend on the different quaternary structure of the two proteins. RelP exists as a homotetramer in solution while Rel_*Seq*_ is a monomer: where helix α1 in RelP is part of the X-ray dimerization interface, the corresponding helix α12 in Rel_*Seq*_ is linked to the C3HB junction domain. Furthermore, the C-terminal portion of RelP (i.e. helixes α6 and α5) is involved in the tetramer formation, while in Rel_*Seq*_ it is linked to the C-terminal regulatory domain.

#### Model Construction

Based on the above considerations, we generated the full chimera model (residues 5–341) using two templates: Rel_*Seq*_ for the HD and C3HB domains, helices α11 and α15 (residues 5–230 and 338–341) and RelP for the SYNTH active site (residues 68–188) complete with its crystallographic ligands (GTP, AMP-CPP, Mg^2+^ and two water molecules) (Supplementary Figure 1). Calculations were performed with the Prime Structure Prediction workflow (Jacobson et al., 2002; Jacobson et al., 2004).

**FIGURE 4:**
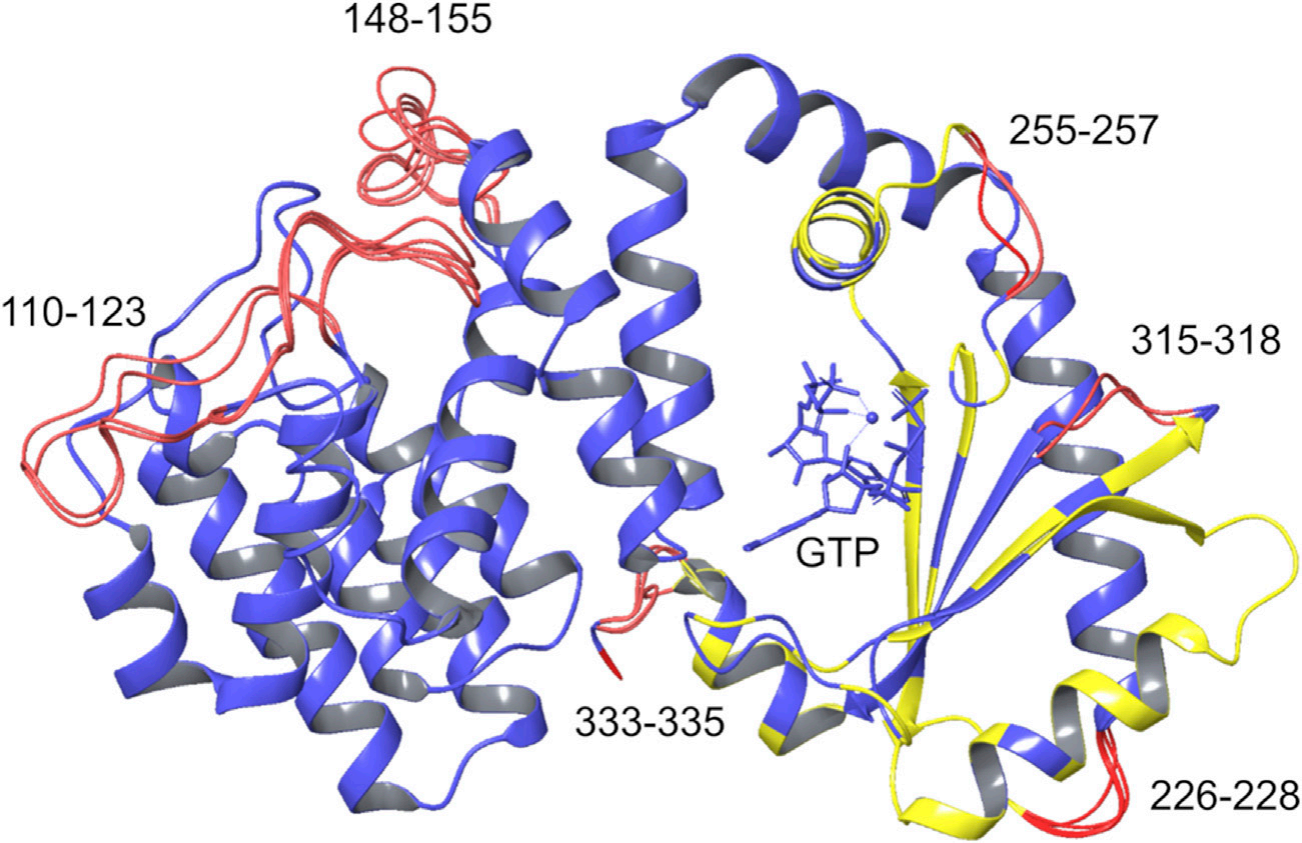
Chimera models generated. The five chimera models generated by homology modeling are overlaid. Blue: coordinates taken from the template; yellow: coordinates taken from the template with optimized side chains; red: rebuilt residues. GTP, AMP-CPP, and the Mg^2+^ ion (blue sphere) are shown within the SYNTH binding site.

The SYNTH domains of Rel_*Seq*_ and RelP were first spatially aligned, i.e. the two crystal structures were re-oriented into a common reference (Figure 3). Secondly, their SYNTH domains sequences (23% of identity) were aligned according to the multiple sequence alignment described by Manav et. al., (Manav et al., 2018) and based on the sequences of RelP and RelQ from different organisms, RelA (from *E. coli*) and Rel_*Seq*_. The 3D model was generated retaining both backbone and side-chain for conserved residues. In the case of non-conserved residues only backbone coordinates were retained, while side-chains were optimized to remove steric clashes and minimized. Missing regions in the template structures (K110-N123 and L152-K159 in Rel_*Seq*_ HD domain) as well as the gaps arising from sequence alignment (T230-Q234, P259-Q261, K319-I322 and Y337-V339 in Rel_*Seq*_ SYNTH domain) were predicted by the knowledge-based method that taps into crystallography databases to find plausible conformations for a given loop sequence. Five 3D models were saved as output by combining different gap predictions and ranked according to the fragment-target sequence similarity and the number of steric clashes, with the first model being the most reliable (Figure 4 and Table 1).

**TABLE 1:** RMSD values calculated on backbone atoms for the five chimera models. The RMSD values calculated on protein backbone atoms (N; C; O; Cα) with respect to model #1 are reported for the SYNTH and HD domain.

Entry	Model	RMSD (Å) of HD Res. 5–155	RMSD (Å) of SYNTH Res. 178–341
1	#1	0	0
2	#2	4.55	0.42
3	#3	0.96	0.52
4	#4	4.47	0.46
5	#5	0.24	0.52

This approach allowed to generate a catalytically competent Rel_*Seq*_ synthetase active site without breaking RelP secondary structural elements and to take into account all the conformational changes induced by AMP-CPP and Mg^2+^ binding. In addition, since the SYNTH domain flanking regions are highly homologue-specific, they were modeled according to the original Rel_*Seq*_ structure.

### Analysis of the Generated Chimera Models

The good quality of the prediction was confirmed for all five models by the very few residues (<1%) populating the disallowed regions of the Ramachandran plot (PROCHEK (Laskowski et al., 1993), Supplementary Figure 2). From a structural point of view, the two HD long gaps showed the higher variability, as indicated by the RMSD values calculated relative to model #1 (Table 1). On the other hand, the conformation of the four short gaps of the SYNTH domain had a little effect on the overall conformation (RMSDs below 0.6 Å, Table1).

Helix α13 represents the part of the SYNTH core that undergoes the largest conformational change to align with the RelP template: the helix maintains its fold but rotates by about 30° relative to Rel_*Seq*_ crystal structure. As expected, the region located between helices α11 and α13 of the SYNTH domain shows some steric clashes, specifically between the side chains of residues I203-Y249 and M207-I245. This issue stems from the procedure adopted, where residues I203 and M207 (α11) were taken from Rel_*Seq*_ without side chains optimization, while I245 and Y249 (α13) were built using the backbone coordinates from RelP and optimizing their side chains. The steric clashes were therefore removed by running a restrained minimization of the heavy-atom displacement (RMSD <0.3 Å) on the whole system.

In order to assess the overall stability of the chimera, we subjected model #1 to a 200 ns MD simulation in NVT conditions (T = 300 K). Analysis of the trajectory showed a twist of the HD relative to the SYNTH domain (Supplementary Figure 3), whereby the domains change their reciprocal orientation while maintaining their tertiary structure. Additionally, the main gap of the HD domain (K110-N123) built by the knowledge-based approach appears to fluctuate considerably, as shown by the root mean square fluctuations (RMSFs) calculated for the protein backbone atoms, which showed the highest peak for residues of this gap (Supplementary Figure 3). We therefore decided to perform an *ab initio* refinement step for this loop in order to generate a different conformation to be used for MD simulations.

### *Ab Initio* Refinement of the K110-N123 Gap

Loop modeling remains one of the most challenging tasks in homology models, especially for loops longer than 10 residues. It is known that knowledge-based prediction accuracy depends on the similarity between the template (from a protein fragment structure database) and the query, while *ab initio* prediction reliability decreases with loop length (Marks and Deane, 2017), but is independent from the database. In our system, the gap length (K110-N123, 14 residues) stretches the accuracy limit of the *ab initio* prediction (Li et al., 2011). In this case, the reliability of both approaches is therefore comparable. We decided to apply the loop refinement protocol available in Prime (Li et al., 2011), generating three *ab initio* conformations for the gap, all displaying a considerably different overall gap conformation (Supplementary Figure 4 and Supplementary Table 1). The model with the lowest energy was selected for MD simulations.

### MD Simulations of the Refined Chimera Model

MD simulations were carried out in NVT (T = 300 K) conditions and explicit water solvent model (TIP3P water model (Jorgensen et al., 1983)) using the Amber 18 package (Case et al., 2018) (AMBER, RRID:SCR_016151). The system was prepared as described in the Materials and Methods section. Amberff14SB force field (Maier et al., 2015) was used for the protein, while for the substrates the AMP-CPP molecule was converted into ATP and the parameters available in the AMBER database were employed (Meagher et al., 2003). Metal ions were treated with a nonbonded model, i.e. an electrostatic plus 12–6 Lennard-Jones (LJ) potential, with an additional dipole-dipole term for Mg^2+^ (Li and Merz, 2014). After system equilibration at 300 K, three independent runs of 400 ns were performed. All the replicas started from the same coordinates file of the equilibrated system, but different velocities were selected. The trajectories were evaluated with respect to protein stability, cluster analysis and ligand-protein interactions.

#### Protein Stability

**FIGURE 5:**
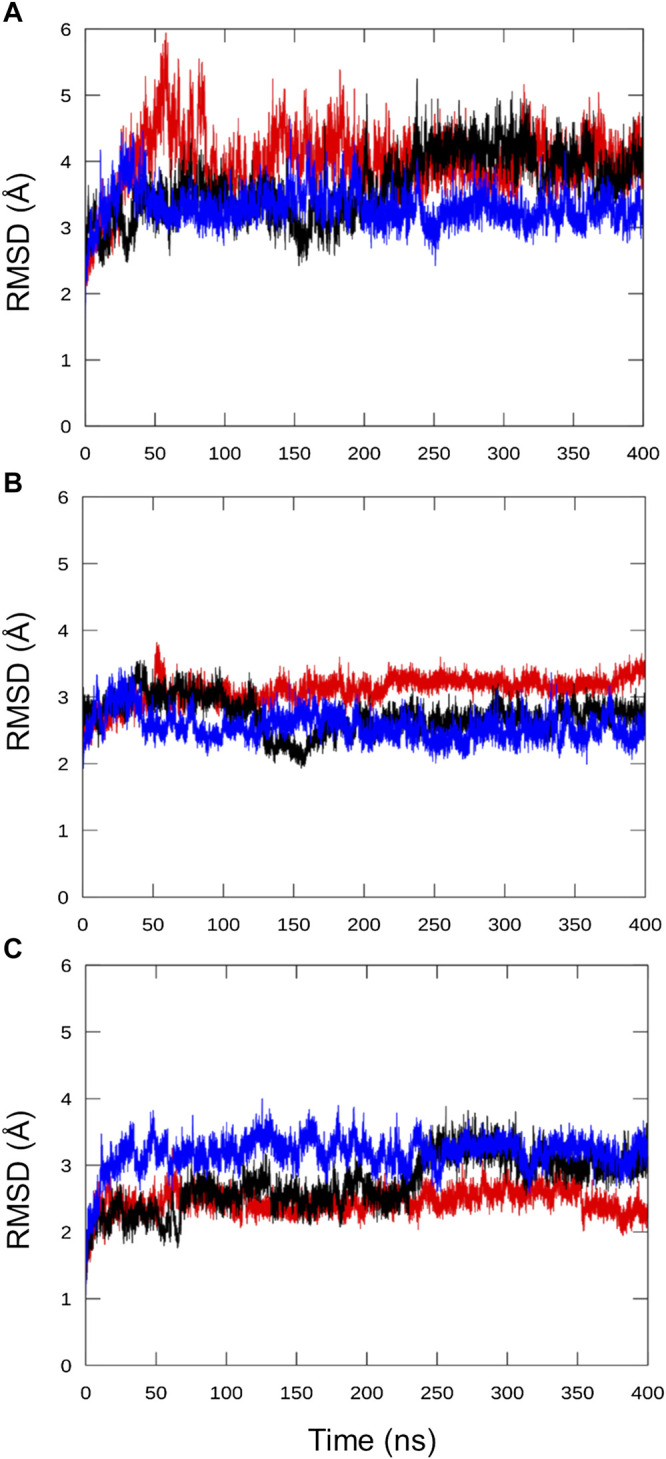
Analysis of the chimera protein stability over the MD simulation time. RMSD was calculated on protein backbone atoms (C, Cα, N, O, H) for the three independent runs with respect to the refined chimera model as a function of the simulation time. **(A)** all residues, **(B)** HD residues alone (5–159) and **(C)** SYNTH residues alone (178–341).

In all replicas, the RMSD, calculated on the protein backbone atoms relative to the input structure, stabilized after about 80 ns. No twist between the HD and SYNTH domains was observed in this case, with the model remaining compact and retaining the SYNTH-HD inter-domain distance observed in Rel_*Seq*_ crystal structure (Figure 5 and Supplementary Table 2).

**FIGURE 6:**
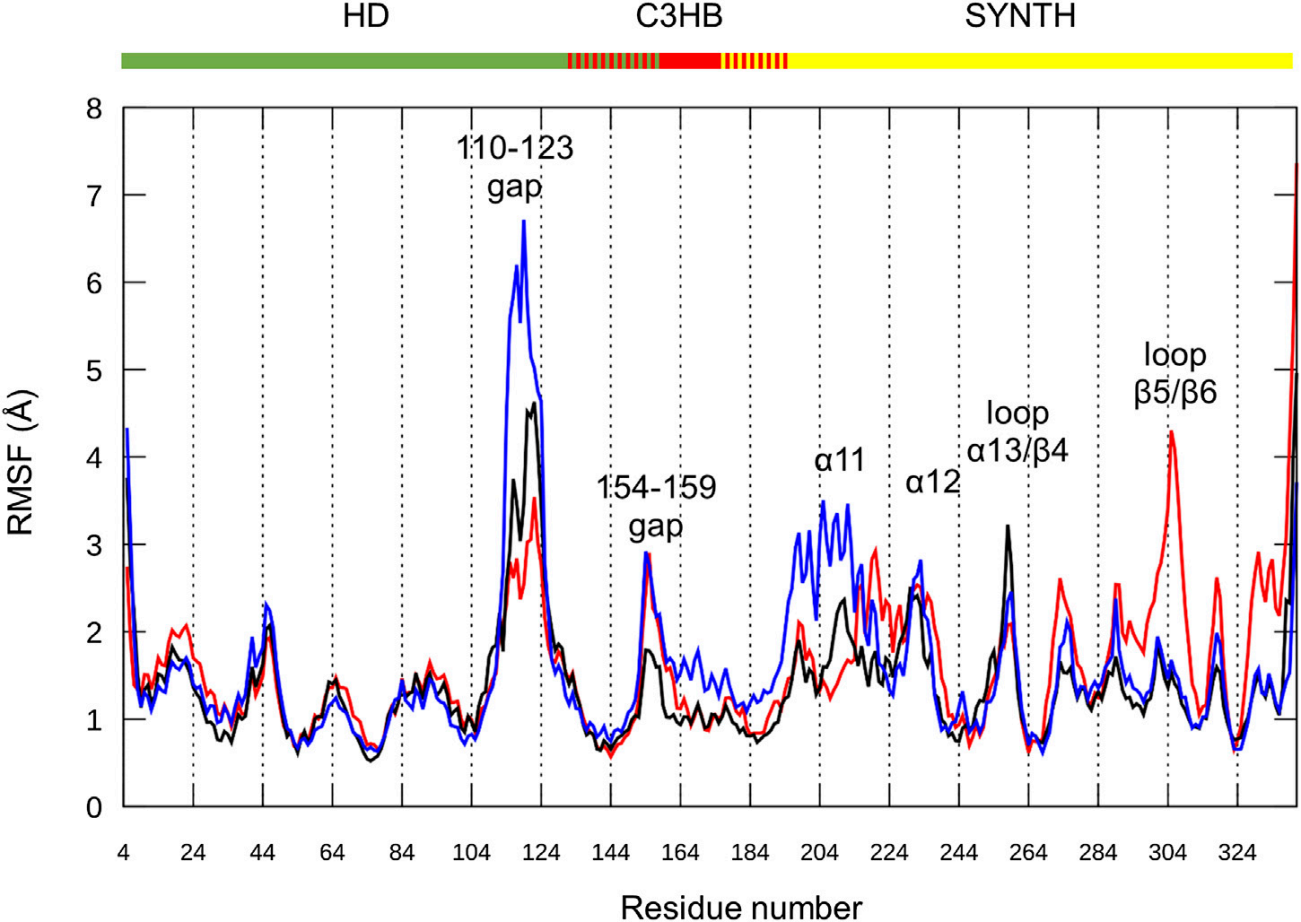
Chimera fluctuation analysis over the MD simulation time. RMSF calculated on protein backbone atoms (C, Cα, N, O, H) in the three replicas (#1 red, #2 black, #3 blue) as a function of the residue number. HD (residues 5–159), C3HB (residues 135–195) and SYNTH (residues 176–341). Peaks with the highest RMSF values are labeled.

As already observed for the unrefined model (Supplementary Figure 3), the RMSF plot for the three replicas show that the greatest variability is once again imparted by the long HD domain loop (residues 110–129, Figure 6). This part of the protein remains highly flexible and the loop interactions observed in the *ab initio* conformation (Supplementary Figure 4) are not maintained. These results suggest that the loop movements are independent of its structure and are probably related to its intrinsically dynamic nature. In support of this hypothesis, this gap remains disordered in the X-ray structure of the Rel_*Seq*_ hydrolase-ON/synthetase-OFF conformation as well (1VJ7. pdb, chain B, residues E112 to S132).

Focusing on the catalytic region, all replicas show flexibility in the loop connecting α12 to β3 (residues 232–235), a portion of the active site not directly in contact with the nucleotides, and part of the loop between α13 and β4 (residues 253–262). The RMSF values of the catalytic residues D264 and E323, as well as those of the residues that should interact with ATP, largely belonging to helix α13, are low, thus indicating a good structural stability. Similarly, the flexible β5/β6 loop interacting with the guanosine ring shows (besides replica #1) low RMSF values for the key residues (Y308, N306, K305 and A335).

The SYNTH domain also shows some flexibility in the region surrounding the active site. In particular, replica #3 (Figure 6, blue line) shows structural flexibility around α11 (residues 197–208), while replica #1 (Figure 6, red line) is less stable around α12 (residues 211–231). During the simulations, α11–α12, as well as the whole SYNTH domain, maintain their global fold, as qualitatively predicted by the content of secondary elements (Supplementary Figure 5), and only the connection between α11 and α12 shows some degree of flexibility. On average, replica #2 (Figure 6, black line) has the lowest RMSF values for the SYNTH domain, indicating a higher stability.

**FIGURE 7:**
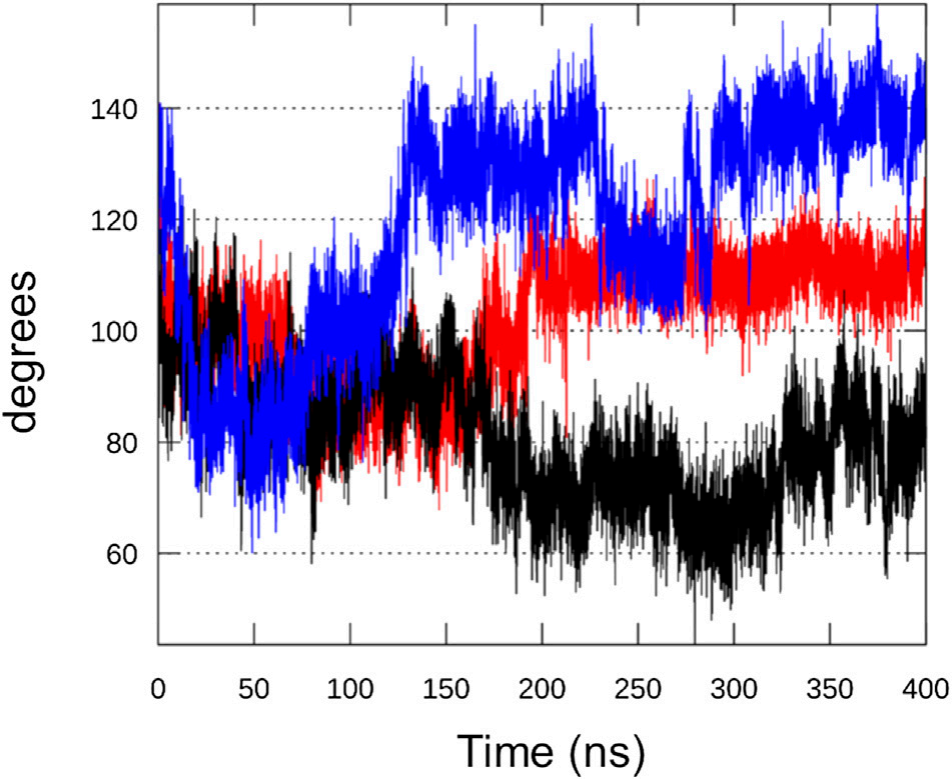
Analysis of Rel_*Seq*_ crystal-modelled interface. Angle formed between helices α11 and α13, the interface between the unaltered SYNTH (from 1vj7. pdb) and the homology modeled portion, during replica#1 (red), #2 (black) and #3 (blue) calculated from the scalar product of the two vectors that run along the two helices.

As already mentioned, helix α13 undergoes a major conformational rearrangement that creates some steric clashes with helix α11. The angle between the two helices in the chimera model (bound to GTP, ATP and Mg^2+^) is 104° while in the Rel_*Seq*_ X-ray structure (GDP alone in the catalytic site) is 77°. We monitored this angle during the simulations, observing that, after oscillating around the input value during the first 10 ns, the angle in replica #1 stabilizes around 110°–120°. In replica #2 it returns to values close to those found in Rel_*Seq*_ X-ray crystal structure (60°–80°), while in replica #3 it settles around higher values (120°–140°) (Figure 7).

More in detail, when we compare the inter-helix angle fluctuations with the same helices RMSD values (backbone atoms) with respect to the input structure, we can observe that the angle fluctuations mainly depend on a movement of helix α11, since α13 is very stable in all replicas (RMSD <1 Å) (Supplementary Figure 6). In comparison, a MD simulation on Rel_*Seq*_ X-ray structure (1VJ7, chain A, GDP alone in the catalytic site) showed that the inter-helix angle relaxes from 77° to around 100° (oscillating between 90° and 110°, data not shown), close to the input value for the chimera model. We therefore deduce that this protein region is flexible and intrinsically dynamic.

#### Cluster Analysis

**FIGURE 8:**
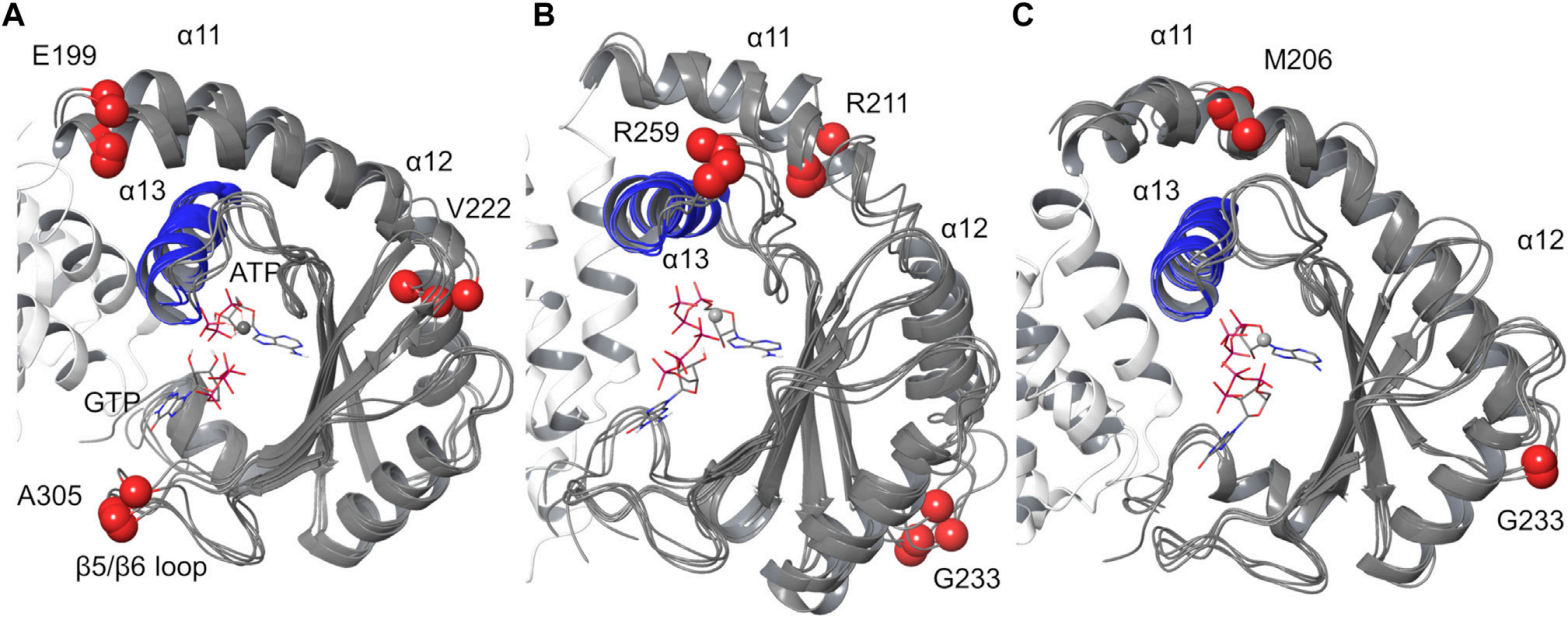
Protein cluster analysis results for the SYNTH domain. Cα atom of residues 197–334 are shown. **(A)** replica #1, **(B)** replica #2, **(C)** replica #3. The representative structures of the main clusters are superimposed to the most populated cluster. RMSD values calculated relative to the most populated cluster c1 < 2 Å (Supplementary Table 3). Blue: helix α13; red spheres: Cα atoms of residues with higher RMSD values.

Cluster analysis was carried out on the SYNTH domain, from α11 to the C-terminal helix (residues 197–337), selecting the protein Cα atoms (average linkage method, *ε* = 1.2). All the replicas display four main clusters with an overall similar conformation (Figure 8, Supplementary Table 3). In agreement with the observations made above, all the clusters show a slightly different orientation of helix α11 with respect to α13, and some degree of flexibility along α11 and α12, where some discontinuities in the helical content have been observed as well (Supplementary Figure 5). Other structural differences are located in the loop regions of the protein (Figure 8).

Cluster analysis performed on ligands’ heavy atoms highlights a single binding mode for ATP corresponding to the input geometry and a conserved binding mode for the guanine ring of GTP, with some variability in the conformations adopted by the triphosphate group (Supplementary Figure 7).

#### Ligand-Protein Interactions

The analysis of the ligand-protein interactions was carried out with respect to the interactions observed in the input chimera model and involving the key and conserved residues of RelP and Rel_*Seq*_ (Supplementary Figures 8–10). The substrate GTP maintained its key π-π interaction with Y308, stabilized by the initial HBs within the binding site. The magnesium ion preserved its coordination sphere during the entire simulation time (for a more detailed description of the interactions see Supplementary Table 4).

In all runs, ATP maintained the folded input conformation and the initial set of interactions with the adenine ring sandwiched between two highly conserved arginine residues, R269 and R241 and the phosphate group coordinating the metal. This peculiar ATP binding mode, proposed on the basis of RelP pre-catalytic structure, was also found for AMP in the crystal structure of the post-catalytic state of Rel_*Tt,*_ where it engages in π -cation interactions with the corresponding arginine residues, R277 and R249 (Supplementary Figure 11). The same substrate conformation and interactions were observed also in the crystal structures of RelP (6FGX.pdb) (Steinchen et al., 2018) and RelQ (5F2V.pdb) (Steinchen et al., 2015) in complex with AMP-CPP alone. To support that a conformational change of the X-ray structure of Rel_*Seq*_ is required to fit the ATP molecule into the SYNTH active site, we carried out docking calculations into such experimental structure*.* Docking poses failed in reproducing the binding mode observed for ATP analogue and AMP in the experimental structures and the adenine ring did not insert between the two arginine residues (Supplementary Figure 12).

For GTP, the binding mode observed for the product in Rel_*Tt*_ is very similar in terms of orientation within the binding site and interactions formed: in both structures the guanine ring forms a conserved π-π interaction with a tyrosine residue (Rel_*Seq*_ Y308, Rel_*Tt*_ Y329) and the phosphate groups are stabilized by the lysine and arginine residues of the pocket (Supplementary Figure 12).

These observations lead us to conclude that the system is stable and capable of retaining both GTP and ATP over time. Moreover, the coordination of ligands inside the catalytic site closely resembles that of Rel_*Tt*_ in its post-catalytic state, including the interactions formed with the key residues of the pocket supporting our hypothesis of a common reaction mechanism for the synthesis of the alarmone by these enzymes.

## Conclusion

Here we reported the first synthetase catalytically competent 3D model of a bifunctional RSH enzyme, Rel_*Seq*_. In the X-ray structure, the synthetase site was only partially occupied by the substrate GDP, lacking the pyrophosphate donor ATP and the Mg^2+^ ion cofactor. Comparative structural analysis of RelP, a small alarmone synthetase, in its pre-catalytic conformation, allowed to identify the specific conformational changes required for Rel_*Seq*_ to become fully competent, i.e. a major shift of helix α13 and a reorientation of the residues required to form the Mg^2+^ ion proper coordination sphere and, ultimately, for the catalysis to take place.

With a homology modeling approach, we generated several full Rel_*Seq*_-RelP chimera models bound to GTP, ATP and Mg^2+^. The longest gap of the structure (residues 110–123), part of a long loop (residues 110–129) of the HD domain, was first rebuilt with a knowledge-based approach and then refined using an *ab initio* approach. The quality of the generated models was assessed and found satisfying (<1% Ramachandran outliers). The steric clashes found at the interface between helices α13 and α11 were resolved with a restrained minimization step. The lowest energy model was selected to carry out MD simulations, necessary to evaluate its stability.

Over the MD runs, the model remained compact, retaining the domains overall fold and the initial SYNTH-HD inter-domain distance. The highest fluctuations were observed at the level of some flexible loops and of helix α11, which relaxes in order to make room for the required reorientation of helix α13. Importantly, all the binding partners (GTP, ATP and Mg^2+^) remained stably anchored to the catalytic site, thus confirming the reliability of the SYNTH site conformation of the model. In particular, ATP is firmly bound to helix α13, further endorsing our initial hypothesis. Noteworthy, our computational work has been further validated by the recently deposited X-ray structure of the post-catalytic conformation of Rel_*Tt*_, a long bifunctional Rel protein.

In conclusion, we have generated a reliable and stable Rel_*Seq*_ model with a catalytically competent synthetase site. This model provides valuable insights into the molecular basis of substrate recognition and is currently being exploited for the design of potential enzyme inhibitors. We envision that finding potent and specific chemical probes targeting this enzyme superfamily will greatly benefit the bacterial persisters research community, helping to dissect their formation mechanism(s) and hopefully providing useful tools in our fight against chronic infections and antimicrobial resistance.

## Materials and Methods

### Templates Preparation for the Homology Modeling

Rel_*Seq*_ X-ray crystal structure (1VJ7. pdb, chain A, hydrolase-OFF/synthetase-ON) was selected as template. It comprises residues 5–341 and a Mn^2+^ ion bound into the HD domain. In the X-ray structure of RelP pre-catalytic state two copies of the same protein (residues 30–194) are arranged as a dimer. Since the two monomers as well as the ligands binding modes are almost identical (RMSD of 0.437 Å calculated on backbone atoms), we then arbitrarily selected chain A for the homology modeling procedure. For residues T100, R152 and L164 showing alternate side chain positions in the pdb structure, the rotamer with the higher average occupancy was selected. In both templates, all crystallographic waters were deleted.

### Molecular Dynamics Preparation and Set up

All the simulations were performed using the AMBER18 package (Case et al., 2018) (AMBER, RRID:SCR_016151) using the ff14SB force field (Maier et al., 2015) and the explicit water solvent (TIP3P model) (Jorgensen et al., 1983). The same starting geometry was employed for all the runs as it corresponds to the homology model #1 with the refined conformation for the K110-N123 loop (i.e. the structure obtained in the loop refinement step corresponding to the lowest Prime energy). The Mn^2+^ ion in the hydrolase domain was maintained to preserve protein stability (it is present in both Rel_*Seq*_ X-ray conformations) and modeled using the default 12–6 Lennard-Jones parameters. For Mg^2+^ ion an additional dipole-dipole term was added according to the 12-6-4 parameters developed by Li and Merz (Li and Merz, 2014). The AMP-CPP molecule was converted to ATP and the available parameters for this nucleotide and for GTP compound were used (Meagher et al., 2003). Both the substrates were in the fully deprotonated state (i.e. a negative charge of −4 and −5 for ATP and GTP molecules, respectively). The two water molecules filling the octahedral coordination around the Mn^2+^ ion were kept and modeled as TIP3P waters. The protonation states of the protein residues were determined using PROPKA (implemented in the Protein Preparation wizard of Maestro v12) (Maestro, RRID:SCR_016748) (Schrödinger, 2018) at pH 7 ± 2. The system was solvated in a cubic simulation box of TIP3P water molecules (15 Å × 15 Å × 15 Å), counter ions (3 Na^+^) were added to neutralize the system and the periodic boundary conditions imposed in the three dimensions. The final simulated system was made of about 83,000 atoms.

After minimizations (2000 steps of steepest descent algorithm first, keeping the complex fixed with a harmonic potential, force constant k = 10 kcal/mol Å^2^, followed by 2000 steps of unrestrained minimization), the system was subjected to an equilibration phase using the sander module of AMBER18. The system was heated at 300 K performing 200 ps (time step, dt = 0.5 ps) at constant volume restraining the complex positions (k = 10 kcal/mol Å^2^). Then a step at constant pressure (*p* = 1 bar, dt = 0.5 ps, 100 ps, T = 300 K) with the Berendsen’s algorithm option for pressure control (relaxation time of 2.0 ps) (Berendsen et al., 1984) was carried out followed by 200 ps of NVT simulation at 300 K with no restraints on the complex. For temperature control, the Langevin thermostat was used (collision frequency of 1 ps^−1^) (Sindhikara et al., 2009). For the electrostatic forces the Particle Mesh Ewald (PME) (Darden et al., 1993) method was applied and a cut-off of 9 Å was used for the non-bonded interactions. All bonds involving hydrogen atoms were constrained using the SHAKE algorithm.

For the production runs, the PMEMD module for GPU was used and three independent replicas of 400 ns (for a total of 3*0.4 μs = 1.2 μs) were run selecting different initial velocities in NVT conditions, (T = 300 K, time step of 2 fs, SHAKE for hydrogen atoms, Langevin thermostat). Replica #1 read velocities and coordinates from the equilibration step while for replica #2 and #3 only coordinates were taken from the last structure of the equilibration step and velocities were randomly chosen on the basis of a Maxwellian distribution at 300 K. For each replica, 20,000 structures were saved for analysis with CPPTRAJ module.

## Data Availability

The raw data supporting the conclusions of this article will be made available by the authors, without undue reservation.
