# Supplementary material for: Homology Model of a Catalytically Competent Bifunctional Rel Protein
Source: Front Mol Biosci. 2021 Feb 3;8:628596. doi: 10.3389/fmolb.2021.628596 (PMC7983052; doi:10.3389/fmolb.2021.628596)
Supplement: Supplementary file 1 [file table1.docx]

| Entry | Model | RMSD (Å) of HD  Res. 5-155 | RMSD (Å) of SYNTH  Res. 178-341 |
| --- | --- | --- | --- |
| 1 | #1 | 0 | 0 |
| 2 | #2 | 4.55 | 0.42 |
| 3 | #3 | 0.96 | 0.52 |
| 4 | #4 | 4.47 | 0.46 |
| 5 | #5 | 0.24 | 0.52 |
