# Supplementary material for: Homology Model of a Catalytically Competent Bifunctional Rel Protein
Source: Front Mol Biosci. 2021 Feb 3;8:628596. doi: 10.3389/fmolb.2021.628596 (PMC7983052; doi:10.3389/fmolb.2021.628596)
Supplement: Supplementary file 2 [file datasheet1.docx]

***Supplementary Material***

**Supplementary Figure 1**


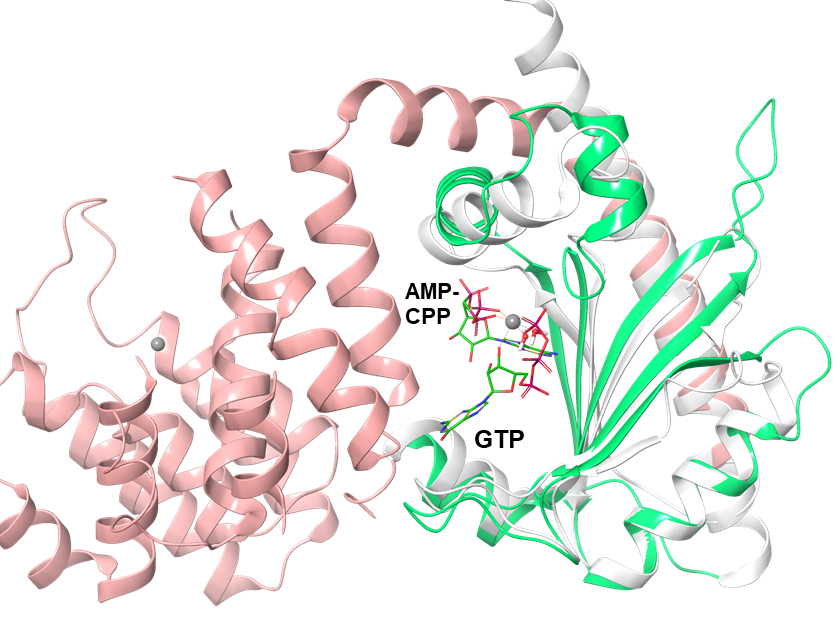


**Figure S1**| **Selection of templates region for the generation of the chimera.** Pink: residues taken from template 1 (Rel*_Seq_*, 5-239 and 338-341); Green: residues taken from template 2 (RelP, 68-188); Grey: residues excluded from the model. For the ligands: GTP (green), AMP-CPP (green), coordination waters (red dots), Mg^2+^ and Mn^2+^ (grey spheres).

**Supplementary Figure 2**


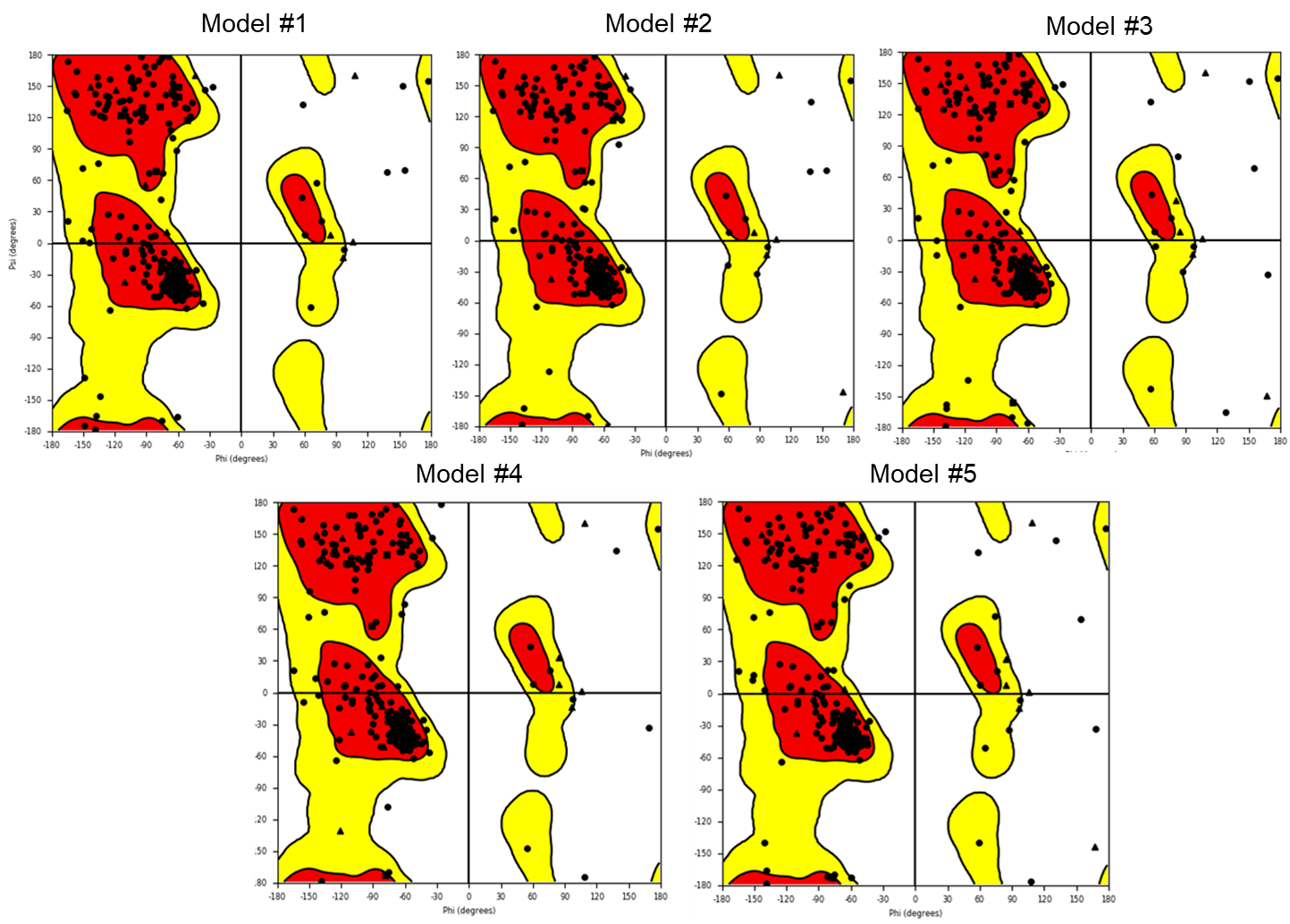


**Figure S2|** Ramachandran plots of the five models generated by the homology modelling procedure.

**Supplementary Figure 3**


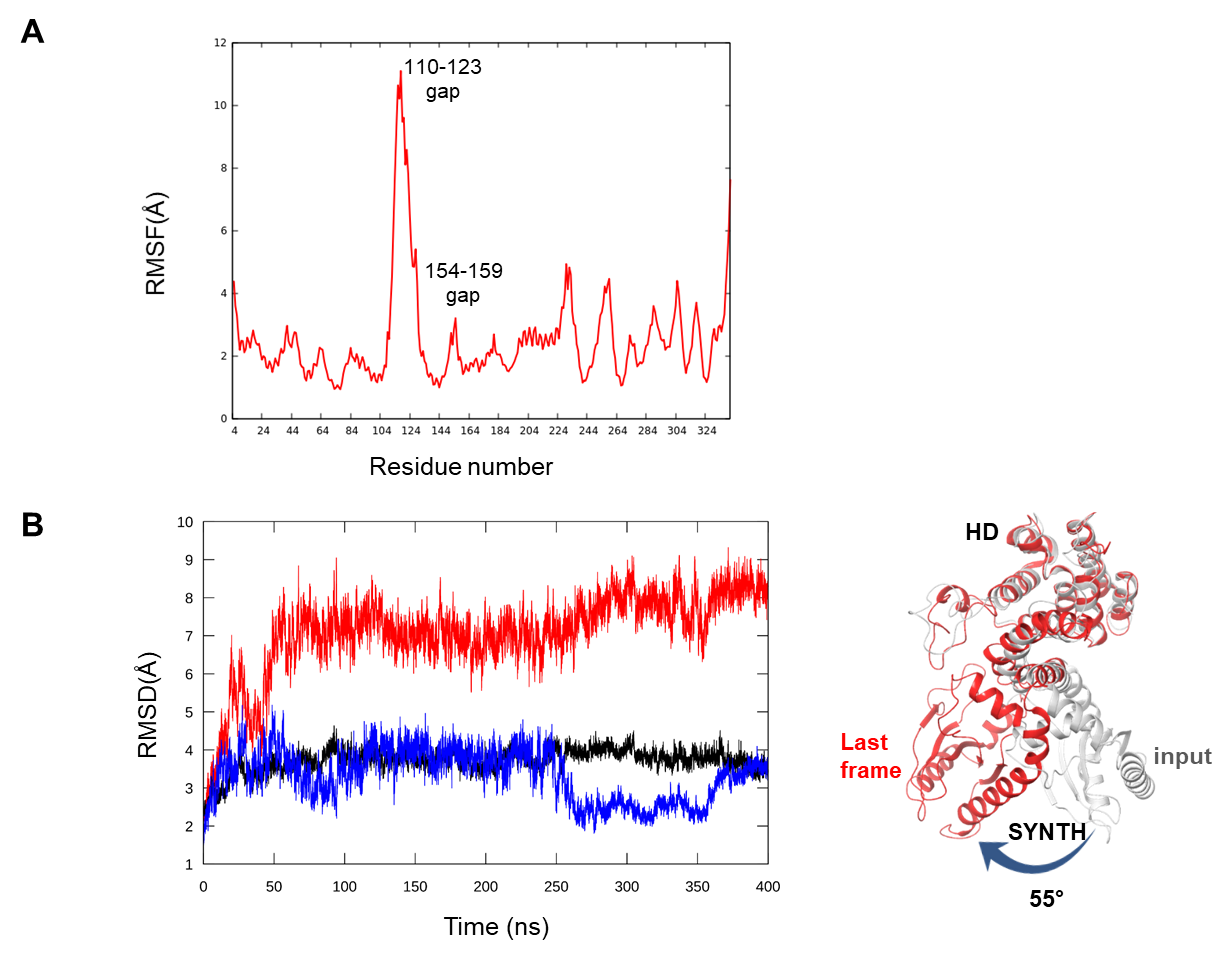


**Figure S3**| **(A)** RMSF and **(B)** RMSD of Model#1 calculated as a function of the simulation time for the protein backbone atoms (C,Cα,N,O,H) during 200 ns of NVT (T=300 K) simulation. **(B)** Reference structure corresponds to the input Model#1. Black, HD (residues 5-159); blue, SYNTH (residues 178-341); red, whole protein (residues 5-341). The tertiary structure of each domain is preserved over the simulation time, while a change in the reciprocal orientation (ca. 55°) of the two domains increases the RMSD values for the whole protein.

The overall fold of each domain is preserved (low RMSD values) while there is an increase of the RMSD value for the whole protein that indicates a change in the reciprocal orientation of the HD and SYNTH domains.

**Supplementary Figure 4**


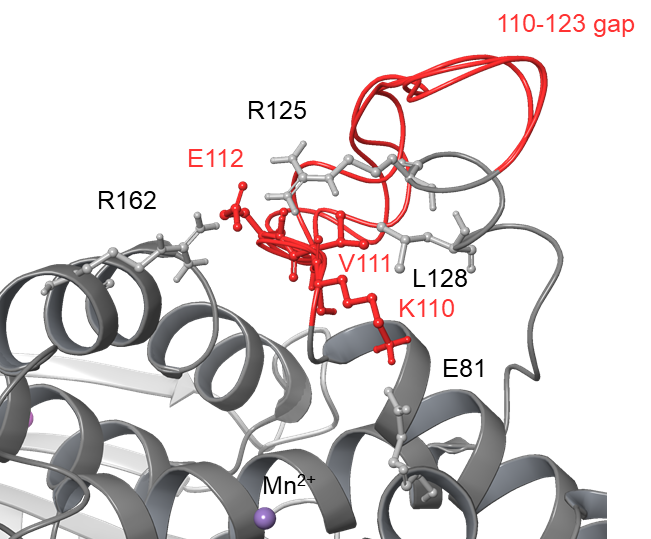


**Figure S4**| The three *ab initio* conformations generated for the K110-N123 gap (red), compared to the knowledge-based protocol (grey). Conformations generated with an ultra-extended method with default option, OPLS_2005 force field (Banks et al., 2005), VSGB implicit water model (Li et al., 2011). Residues involved in key interactions are labelled.

All *ab initio* structures display a considerably different overall gap conformation, showing a salt bridge between the loop residue K110 and the side chains of E81 of the HD domain, an intra-loop hydrophobic interaction between V111 and L128 side chains, and salt bridges between E112 side chain and R162 (C3HB domain) and R125 of the loop.

**Supplementary table 1**

**Table S1**| Distance between the centre of mass of the HD and SYNTH domains (d) and gyration radius (Rg) calculated on protein Cα during the three independent runs.

| Entry | Replica | HD-SYNTH d (Å) | R_g_(Å) |
| --- | --- | --- | --- |
| 1 | Rel*_Seq_* 1VJ7 | 33.3 | 22.5 |
| 2 | #1 | 33.8 ± 0.7 | 23.3 ± 0.3 |
| 3 | #2 | 32.3 ± 0.7 | 22.7 ± 0.2 |
| 4 | #3 | 32.6 ± 0.5 | 22.9 ± 0.2 |

**Supplementary table 2**

**Table S2|** RMSD calculated on backbone atoms for the 110-123 gap for each model with respect to model #1. Models ranked according to the Prime energy with respect to the lowest energy structure.

| **Entry** | **Model** | **ΔE(kcal/mol)** | **RMSD (Å)**  **Res.110-123** |
| --- | --- | --- | --- |
| 1 | #1 | // | 0 |
| 2 | #2 | 0.08 | 1.97 |
| 3 | #3 | 0.362 | 2.125 |
| 4 | Knowledge based |  | 3.82 |

**Supplementary Figure 5**


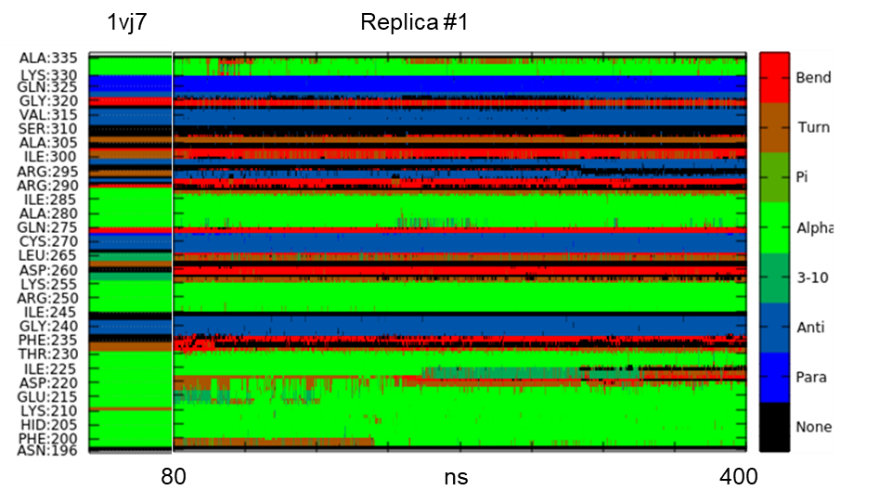


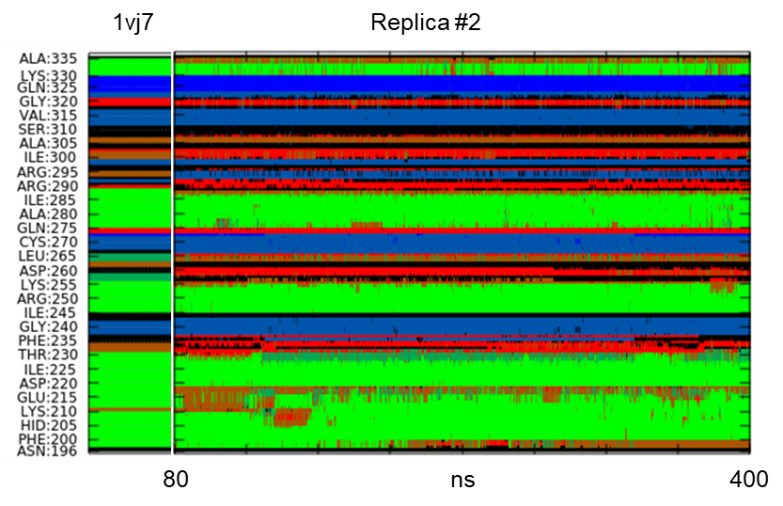


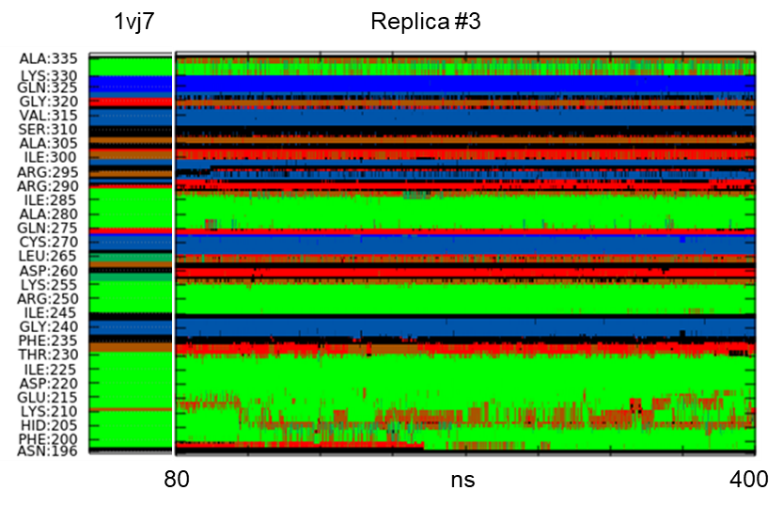


**Figure S5**| Secondary structure analysis performed for all the replicas starting from 80 ns on the SYNTH domain (residues 196-335). The analysis of 1vj7.pdb (chain A) is shown on the left for comparison.

The region around α11- α12 corresponds to an unsolved portion of the Rel*_Seq_* Synthetase-OFF conformation (chain B, residues 210-217), indicating that flexibility for this portion of the protein is plausible.

**Supplementary Figure 6**


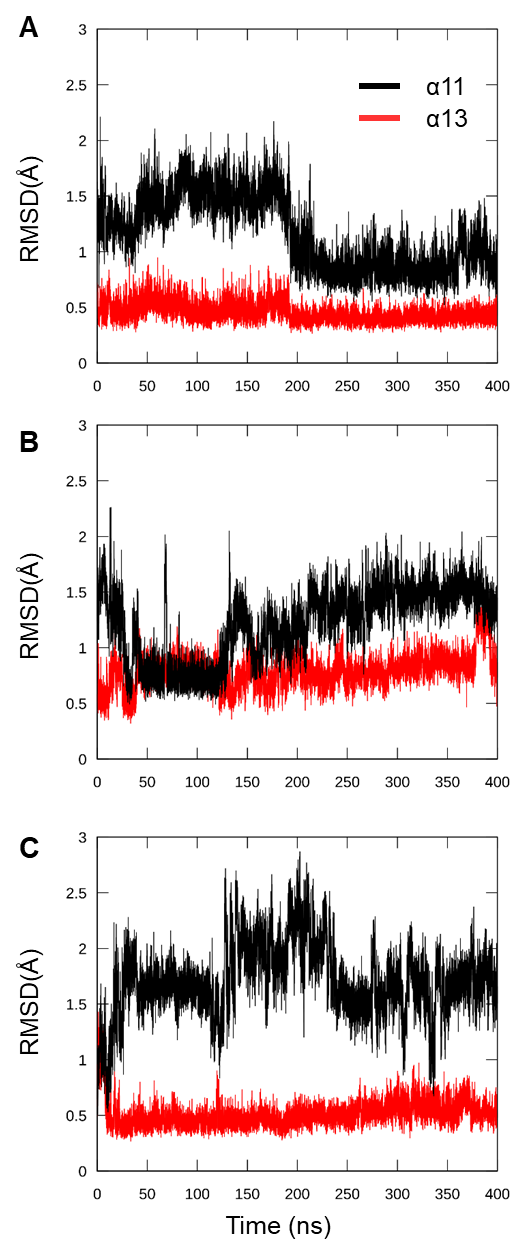


**Figure S6**| RMSD values of backbone atoms calculated for α11 (black, residues 197-209) and α13 (red, residues 245-255) with respect to the input structure. **(A)** replica #1; **(B)** replica #2; **(C)** replica #3.

**Supplementary Table 3**

**Table S3|** **Cluster analysis of the SYNTH domain for the three replicas**. Cluster populations (percentage, pop) and RMSD values calculated on Cα atoms of residues 197-337 with respect to the input structure are reported. Only cluster with pop > 5% are considered.

| **cluster** | **replica #1**  **Pop (%) RMSD(Å)**  input c1 | | | **replica #2**  **Pop (%) RMSD(Å)**  input c1 | | | **replica #3**  **Pop (%) RMSD(Å)**  input c1 | | |
| --- | --- | --- | --- | --- | --- | --- | --- | --- | --- |
| **c1** | 40 | 3.10 | // | 33 | 2.40 | // | 23 | 2.24 | // |
| **c2** | 19 | 3.10 | 1.10 | 11 | 2.91 | 1.97 | 17 | 2.26 | 0.88 |
| **c3** | 11 | 2.80 | 1.64 | 10 | 2.79 | 1.53 | 12 | 2.53 | 0.96 |
| **c4** | 7 | 2.68 | 1.73 | 9 | 2.67 | 1.11 | 11 | 2.30 | 1.59 |

**Supplementary Figure 7**


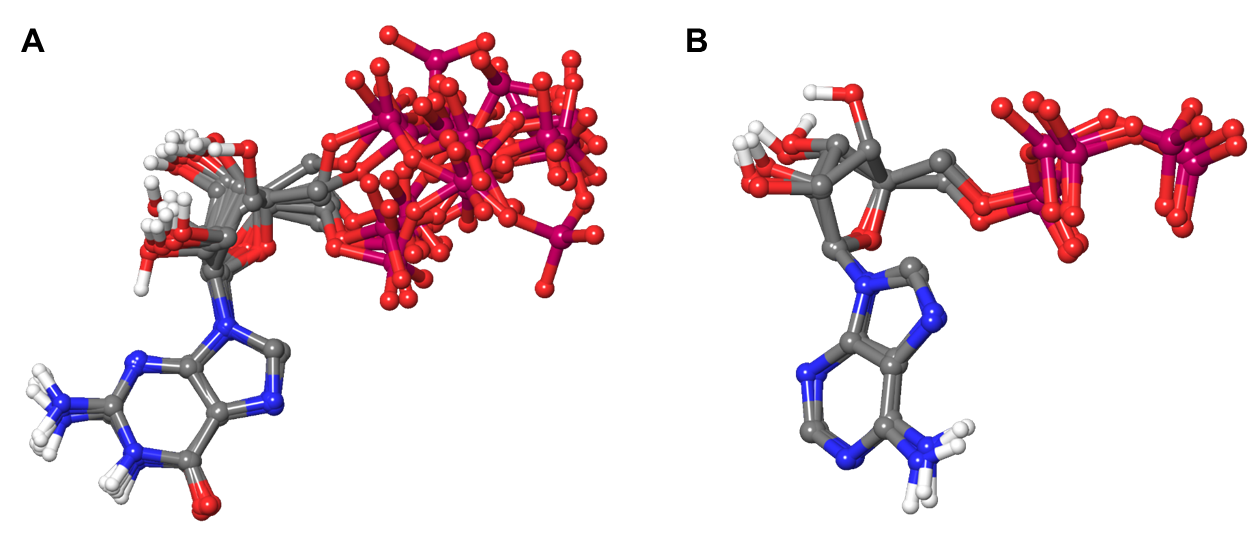


**Figure S7| Ligand clusters analysis results** (averagelinkage, ε = 1, ligand heavy atoms). **A)** the most representative structures of GTP molecules are shown; replica #1 has 4 main clusters, #2 has 2 clusters, #3 has 3 clusters. **B)** A single cluster was found for ATP in each replica.

**Ligand-protein interactions in the X-ray structures**

For each ligand, the details of the interactions formed in the RelP pre-catalytic state and Rel_Seq_ crystal structures are reported below.

**Supplementary Figure 8**

*GDP/GTP.* The binding modes of the substrates, GTP for RelP and GDP for Rel_Seq_, are superimposable, as both guanosine rings form a π-π interaction with a conserved tyrosine (Y151 in RelP, Y308 in Rel_Seq_ ) and hydrogen bonds with the residues of the pocket (N149 and E189 in RelP; N306 and A335 in Rel_Seq_). In both active sites, there are basic residues, largely conserved in SAS/Rel enzymes, for the interaction with the phosphates. In RelP, the additional γ-phosphate group of GTP points toward the metal ion. Other conserved residues of the pocket (Rel_Seq_-H312/RelP-H155, Rel_Seq_-Y299/RelP-Y142) form hydrogen bonds with the phosphates. The ribose 3’-OH of GTP is coordinated to Mg^2+^.


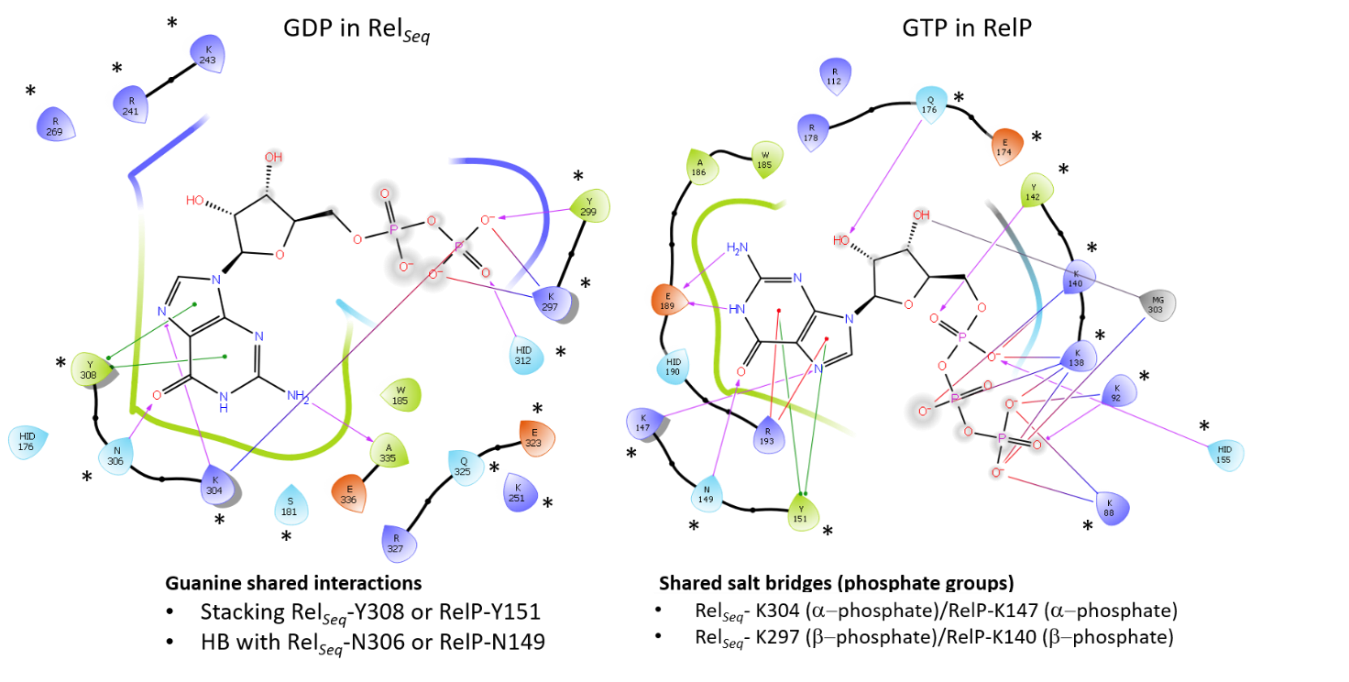


**Figure S8| 2D ligand interactions diagram of GDP in the X-ray structure of Rel*_Seq_* (left, 1vj7.pdb chain A) and GTP in the pre-catalytic state of RelP (right, 6ewz.pdb).** Green lines: π-π interactions; red lines: π-cation interactions; purple arrows: hydrogen bonds; red-to-blue lines: salt bridges. *Conserved residues.

**Supplementary Figure 9**

*Metal coordination*. In the pre-catalytic state of RelP, Mg^2+^ directly coordinates the ribose 3’-OH of GTP, two oxygen atoms of ATP γ/β-phosphate moieties and the side chain of D107. Two water molecules, one of which mediates the interaction with E174, complete the octahedral coordination of the metal.


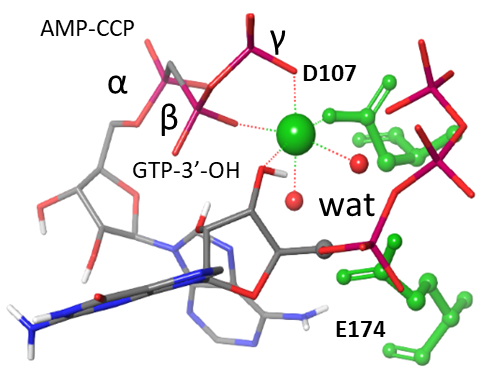


**Figure S9**| **Mg^2+^ octahedral coordination in the RelP pre-catalytic state.** Green: catalytic residues (E174 and D107); red dots: water molecules; green sphere: Mg^2+^.

**Supplementary Figure 10**

*ATP analogue.* In RelP, the AMP-CPP molecule adopts a U-shaped conformation with the adenine ring forming π-cation interactions with R78 (β1) and R112 (β2), conserved in Rel_Seq_ (R241-β3 and R269-β4), and a hydrogen bond between adenine NH2 and the side chain and backbone-C=O of E174 (corresponding to E323 in Rel_Seq_). The β- and γ-phosphates are coordinated to the metal ion and form salt bridges with basic residues of the pocket (K80, K88, R78 and R91), also conserved in Rel_Seq_ (R241, K243, K251). These residues belong to helix α2 (α13 in Rel_Seq_), the part of the catalytic core that diverges the most between the two structures. There is a hydrogen bond between a α-phosphate oxygen and the side chain of S84 (also conserved in Rel_Seq_, S247). The sugar moiety is in contact with the arginine residue R78 and R112.


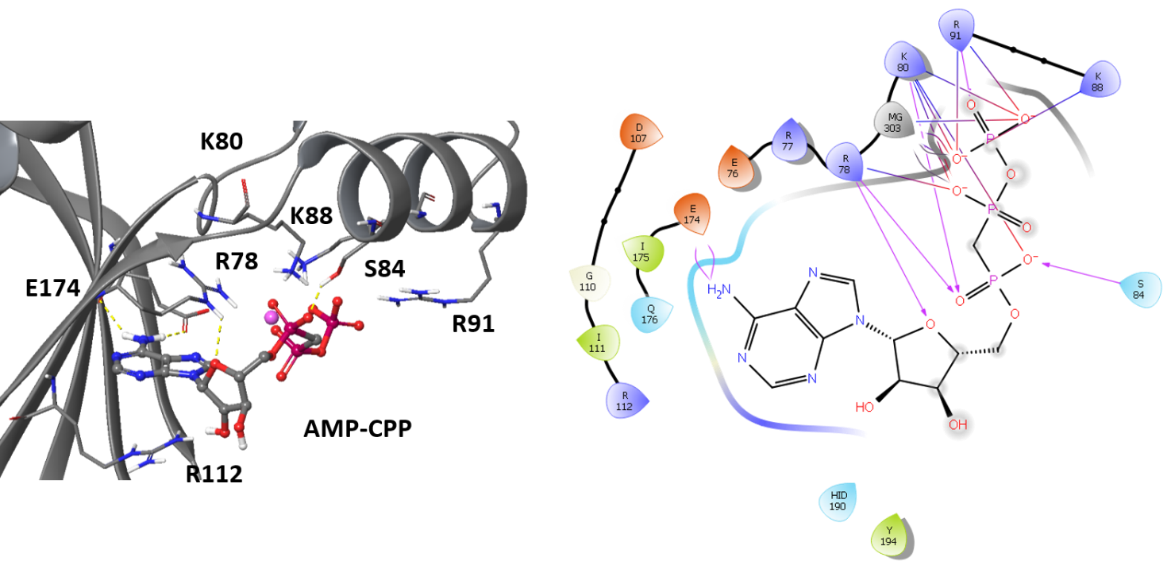


**Figure S10| AMP-CPP binding mode in the X-ray structure of RelP pre-catalytic state (6ewz.pdb) and its 2D interactions diagram (right).** The most relevant residues of the binding pocket are shown.

**Supplementary Figure 11**


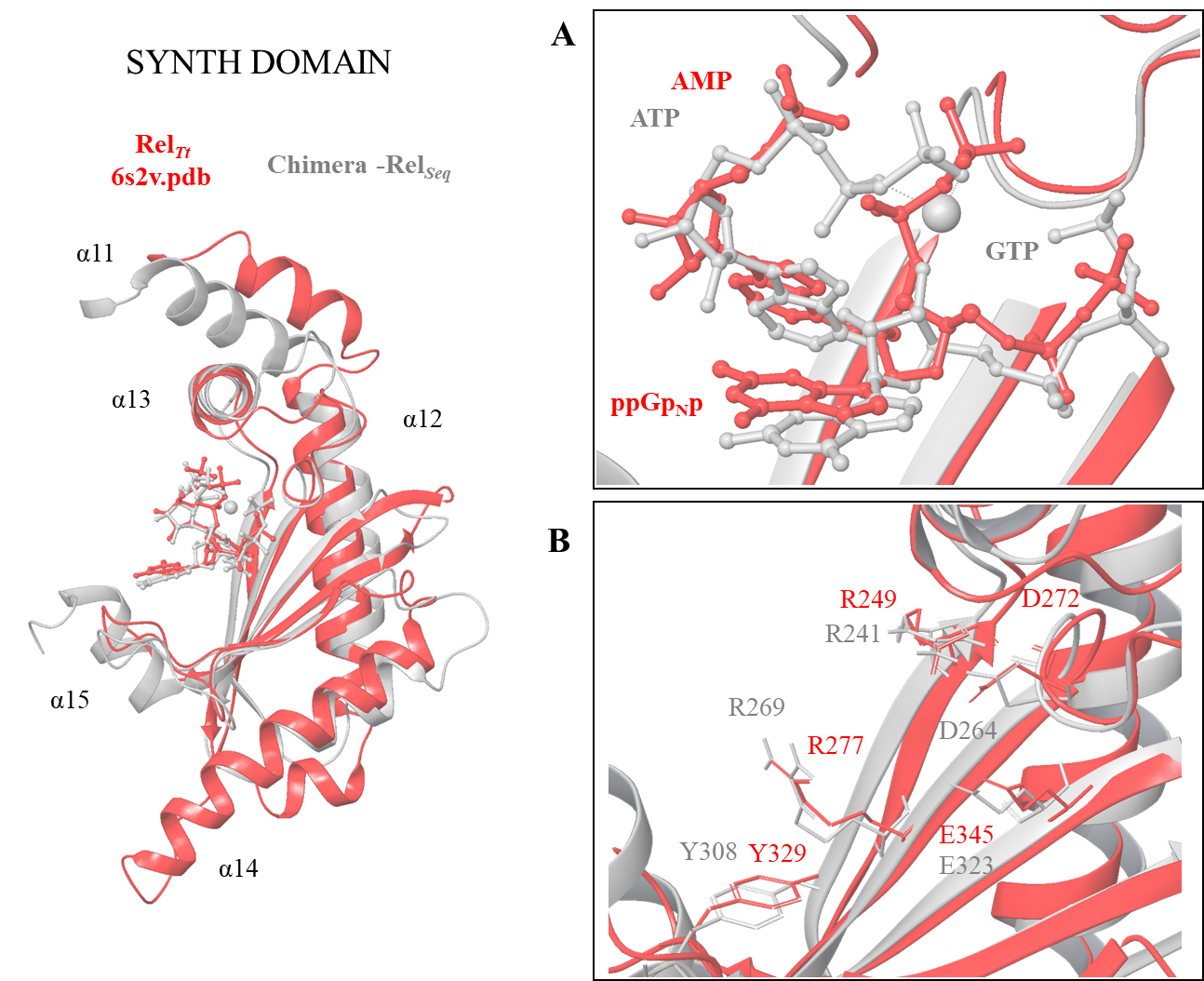


**Figure S11| X-ray structure of the open state of Rel*_Tt_* (6s2u.pdb, red) superimposed to Rel_Seq_ chimera model (grey).** Only the SYNTH domain is shown (left) with a focus on the ligands binding into the active site (right, A) and on the key residues of the catalytic site (right, B).

**Supplementary Figure 12**


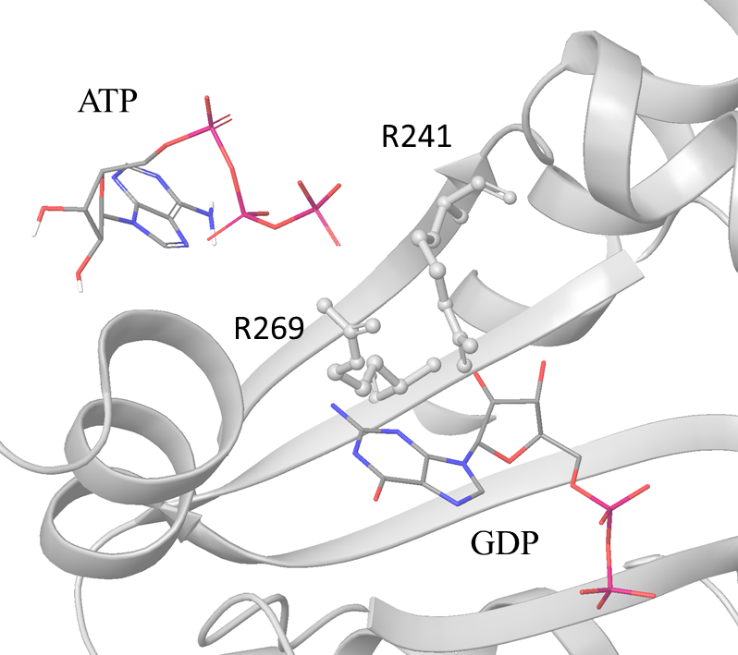


**Figure S12| Docking best pose of ATP into the Rel_Seq_ crystal structure (1vj7.pdb, chain A).** The arginine residues used to center the grid (OPLS3, inner box=10 Å, outer box of 20 Å) are highlighted. Docking calculation were performed using GLIDE software (Schrödinger Release 2018-1: Glide, Schrödinger, LLC, New York, NY, 2018) in Standard Precision (SP). The U-shaped conformation of ATP found in the RelP crystal was rigidly docked (OPLS3, 10 poses were saved).

**Analysis of Ligand-protein interactions during the MD simulations**

The analysis of the ligand-protein interactions was carried out with respect to the interactions observed in the input chimera model and involving the key and conserved residues of RelP and Rel_Seq_. In all the runs the ATP ligand maintained the key π-cation interactions with R241 (β3) and R269 (β4) (d < 5.5 Å), a stable salt bridge of the α-phosphate with R241 and the coordination of the β,γ-phosphate to the metal ion (Mg^2+^). The hydrogen bond (HB) between the side chain of S247 (α13) and the α-phosphate group was maintained along with the HB between the backbone of I269 (β4) and the Adenine-N6 while the HB between this amino group and the E323 side chain was less conserved (Suppl. Table 4). In replicas #2 and #3 a new HB between the β-phosphate and the side chain of W185 (α10) was formed. The triphosphate group is stably in contact with the basic residues of helix α13. Indeed, salt bridges with K243 (α,β-phosphates) and K251 (γ- phosphate) populated >75% of all the replicas. These observations are in agreement with the cluster analysis results.

On the other hand, GTP the starting π-π interaction with Y308 side chain was maintained in all the replicas (distance < 5Å) and further stabilized by the HBs with N306 side chain and the backbone of A335 (Suppl. Table 4). A major variability was observed for the interaction of the more flexible triphosphate group that, during MD runs, formed unstable salt bridges with the lysine and arginine residues of the pocket, in a sort of statistical rebinding (e.g. α-phosphate alternates between K255, K297 and K304).

Finally, we analysed the coordination sphere of the metal co-factor (Mg^2+^). The ion interacts with E323-Cδ, D264-Cγ and two water molecules (WAT-Ow) at a distance < 3.5 Å; it is coordinated by the ATP β,γ-phosphate groups and the distance from GTP 3’-OH remains close to the input value (3.2 Å), stabilizing after 80ns around a value of 3.9 ± 0.3 Å.

**Supplementary Table 4**

**Table S4|** **Most populated ligand-protein interactions.** Percentages were calculated for each replica (#1, #2, #3) considering the distance between ATP/GTP atoms (labelled with *) and the protein residues (atom explicitly indicated). (a) distance between atoms < 4 Å; (b) distance between atoms < 5 Å. sc: side chain; bb: backbone;

| **Entry** | **ATP atom** | **Residues** | **Type of interaction** | **% structures** | | |
| --- | --- | --- | --- | --- | --- | --- |
|  |  |  |  | **#1** | **#2** | **#3** |
| 1 | Adenine ring | R241sc-CZ  R269sc-CZ | **π-cation** | 100  100 | 100  100 | 100  100 |
| 2 | β,γ-P*O_4_^-^ | Mg^2+^ | **Metal-coordination** | 100 | 100 | 100 |
| 3 | Adenine ring C6-N*H_2_ | I269bb-O | **HB^(a)^** | 96 | 90 | 99 |
| 4 | Adenine ring C6-N*H_2_ | E323sc-Cδ | **HB^(a)^** | 38 | 19 | 28 |
| 5 | α-P*O_4_^-^ | S247sc-HG | **HB^(a)^** | 85 | 89 | 96 |
| 6 | β-P*O_4_^-^ | W185sc-N_ε1_ | **HB^(a)^** | - | 63 | 81 |
| 7 | γ- P*O_4_^-^ | K251-NZ | **salt-bridge^(b)^** | 100 | 100 | 100 |
| 8 | α-P*O_4_^-^ | K243-NZ | **salt-bridge^(b)^** | 100 | 100 | 100 |
| 9 | β- P*O_4_^-^ | K243-NZ | **salt-bridge^(b)^** | 73 | 97 | 93 |

| **Entry** | **GTP atom** | **Residues** | **Type of interaction** | **% structures** | | |
| --- | --- | --- | --- | --- | --- | --- |
|  |  |  |  | **#1** | **#2** | **#3** |
| 1 | Guanine ring | Y308sc | **π-cation** | 100 | 100 | 100 |
| 2 | Guanine ring C6=O* | N306sc-ND2 | **HB^(a)^** | 93 | 99 | 94 |
| 3 | Guanine ring N2* | A335bb-O* | **HB^(a)^** | 67 | 99 | 99 |
| 4 | 3’-O*H | Mg^2+^ | **Metal-coordination** | 100 | 100 | 100 |

**References**

Banks, J.L., Beard, H.S., Cao, Y., Cho, A.E., Damm, W., Farid, R., et al. (2005). Integrated Modeling Program, Applied Chemical Theory (IMPACT). *J Comput Chem* 26(16)**,** 1752-1780. doi: 10.1002/jcc.20292.

Li, J., Abel, R., Zhu, K., Cao, Y., Zhao, S., and Friesner, R.A. (2011). The VSGB 2.0 model: a next generation energy model for high resolution protein structure modeling. *Proteins* 79(10)**,** 2794-2812. doi: 10.1002/prot.23106.
